# The Proteomic Composition and Organization of Constitutive Heterochromatin in Mouse Tissues

**DOI:** 10.3390/cells13020139

**Published:** 2024-01-11

**Authors:** Annika Schmidt, Hui Zhang, Stephanie Schmitt, Cathia Rausch, Oliver Popp, Jiaxuan Chen, Dusan Cmarko, Falk Butter, Gunnar Dittmar, Frederik Lermyte, M. Cristina Cardoso

**Affiliations:** 1 Cell Biology and Epigenetics, Department of Biology, Technical University of Darmstadt, 64287 Darmstadt, Germanystephanieschmitt0801@gmail.com (S.S.);; 2 Proteomics Platform, Max-Delbrueck-Center for Molecular Medicine in the Helmholtz Association, 13125 Berlin, Germany; 3 Institute of Molecular Biology (IMB), 55128 Mainz, Germany; 4 Institute of Biology and Medical Genetics, First Faculty of Medicine, Charles University and General University Hospital in Prague, 128 00 Prague, Czech Republic; 5 Clemens-Schöpf Institute of Organic Chemistry and Biochemistry, Department of Chemistry, Technical University of Darmstadt, 64287 Darmstadt, Germany

**Keywords:** brain, heterochromatin, immunofluorescence staining, liver, quantitative mass spectrometry, proteomics

## Abstract

Pericentric heterochromatin (PCH) forms spatio-temporarily distinct compartments and affects chromosome organization and stability. Albeit some of its components are known, an elucidation of its proteome and how it differs between tissues in vivo is lacking. Here, we find that PCH compartments are dynamically organized in a tissue-specific manner, possibly reflecting compositional differences. As the mouse brain and liver exhibit very different PCH architecture, we isolated native PCH fractions from these tissues, analyzed their protein compositions using quantitative mass spectrometry, and compared them to identify common and tissue-specific PCH proteins. In addition to heterochromatin-enriched proteins, the PCH proteome includes RNA/transcription and membrane-related proteins, which showed lower abundance than PCH-enriched proteins. Thus, we applied a cut-off of PCH-unspecific candidates based on their abundance and validated PCH-enriched proteins. Amongst the hits, MeCP2 was classified into brain PCH-enriched proteins, while linker histone H1 was not. We found that H1 and MeCP2 compete to bind to PCH and regulate PCH organization in opposite ways. Altogether, our workflow of unbiased PCH isolation, quantitative mass spectrometry, and validation-based analysis allowed the identification of proteins that are common and tissue-specifically enriched at PCH. Further investigation of selected hits revealed their opposing role in heterochromatin higher-order architecture in vivo.

## 1. Introduction

The DNA in the cell nucleus is not packed uniformly and chromatin can be broadly subdivided into two cytologically different types, the more open euchromatin and the highly compacted heterochromatin [1,2]. Euchromatin contains the majority of actively expressed genes, whereas heterochromatin comprises mainly inactive genes and various repeat elements. The latter is further characterized by high cytosine methylation levels and methyl-CpG binding proteins, as well as specific histone modifications like H3K9 trimethylation and proteins recognizing these modifications [3]. Heterochromatin can be subdivided into facultative and constitutive heterochromatin [4]. Facultative heterochromatin can become transcriptionally active in different settings as upon cell differentiation or during development [3,5]. Constitutive heterochromatin is considered more stable occurring at the same genetic regions in different cell types [6] and it forms mainly at (peri)centromeric regions and telomeres containing tandem repetitive sequences [3,6]. In mouse cells, major satellite repeats are located at the pericentromeric and minor satellite sequences in the centromeric region of chromosomes [7,8,9]. In the interphase, the pericentromeric satellite DNA from different chromosomes clusters to form pericentromeric heterochromatin compartments (PCH, also called chromocenters) ([10], reviewed in [11]). Several studies highlight the involvement of PCH in the subnuclear organization of chromosomes [12] during the cell cycle [13], development [14], differentiation [15,16,17], and disease [18,19,20,21]. Although pericentromeric DNA satellite repeat sequences were first considered to be non-functional, several studies reported possible functions of transcription of constitutive heterochromatin (reviewed in [6,22]). Its transcription was described in various contexts, including proliferating cells [23], cellular differentiation [24,25,26,27,28], cellular senescence [29], cellular stress [22,30,31,32,33], and diseases like cancer [29,34,35,36]. Heterochromatin malfunction was also reported to lead to genome instability by inducing aberrant repair of repeat elements, replication stress, transposon activation, and errors in chromosome segregation (reviewed in [37]). Although pericentromeric regions are evolutionarily conserved, the DNA tandem repeats show variations in sequence and length between species (reviewed in [38]). The PCH compartment number was reported to differ between cell types [39,40,41], change during development [14], upon cellular differentiation [15,16,17], or by changes in the levels of certain proteins [15].

Although, as mentioned above, several studies deal with PCH compartmentalization and functions, much less is known about their protein composition. In addition, the very different organization of PCH compartments in different tissues raises the question of whether these differences reflect their distinct protein composition. Here, we tested the hypothesis that the distinct PCH compartment organization might originate from a differing quantitative proteomic composition. Thus, we analyzed the heterochromatin proteome from mouse tissues (ex vivo) in an unbiased manner by adapting a protocol for PCH isolation based on a series of sucrose gradient centrifugations initially described by Prusov and Zatsepina [42,43] and performing quantitative mass spectrometry analysis. The analysis of PCH fractions and whole nuclei resulted in the identification of common PCH proteins, but also quantitative differences in PCH protein abundance between the tissues. However, large amounts of general chromatin proteins as well as nuclear membrane- and RNA-related proteins, which do not locally accumulate at PCH regions, were also identified. Further comparative analysis of mass spectrometry data indicated that these proteins had a much lower abundance in PCH. Hence, a protein abundance-based strategy was adopted to more strictly identify PCH protein candidates that were validated by further analysis. Importantly, the methylcytosine-binding protein MeCP2 was recognized to be prominent in brain PCH by comparing the protein abundance in different PCH fractions, while histone H1 was more prominent in liver PCH. Finally, we found that MeCP2 and H1 compete with each other for binding at PCH regions and regulate PCH organization in opposite directions.

## 2. Materials and Methods

### 2.1. Organ Preparation

For nuclei and subsequent PCH isolation, 3-month-old C57BL/6 mice (Charles River Laboratories, Inc., Sulzfeld, Germany) were sacrificed according to the animal care and use regulations (Government of Hessen, Darmstadt, Germany), and the organs of interest were collected, washed with PBS, and frozen in liquid nitrogen.

The organs used for tissue sections and subsequent immunofluorescence staining were isolated as described above and fixed in 10% buffered formalin solution (#HT501128, Sigma-Aldrich, St. Louis, MO, USA) for 24 h. Tissues were sequentially dehydrated in 70% ethanol (#970, Richter Chemie GmbH, Nerdlen, Germany) for 30 min, 70% ethanol for 45 min, 96% ethanol for 60 min, and 96% ethanol for 45 min, twice in absolute ethanol for 45 min followed by xylol for 60 min and 30 min. The organs were embedded in paraffin (#CN49.2, Carl Roth, Karlsruhe, Germany), transferred to embedding cassettes, cooled down slowly, and sliced using a microtome into 6 μm thick slices.

### 2.2. Isolation of PCH from Mouse Organs

All steps for nuclei and PCH isolation were performed with solutions precooled on ice. For nuclei isolation, the frozen mouse brains were crushed to powder and homogenized in 15 mL 0.25 M sucrose (#4661.2, Carl Roth) solution in buffer A (20 mM triethanolamine–HCl (pH 7.6) (#T1377, Sigma-Aldrich Chemie GmbH (Merck), Munich, Germany), 30 mM KCl (#H1758, Sigma-Aldrich Chemie GmbH (Merck)), 10 mM MgCl2 (#M0250, Sigma-Aldrich Chemie GmbH (Merck)), 1 mM dithiothreitol (DTT)(#04010, Sigma-Aldrich Chemie GmbH (Merck)), 1 mM phenylmethylsulfonyl fluoride (PMSF) (#6367.1, Carl Roth)). After centrifugation for 10 min at 1000× *g*, the supernatant was discarded, and the pellet was resuspended in 2.5 M sucrose buffer (2.5 M sucrose in buffer A) to a final sucrose concentration of 2.1 M. The raw nuclei fraction was obtained by centrifugation for 30 min at 50,000× *g* using an SW28 rotor (swinging bucket rotor SW28, Beckman Coulter, Brea, CA, USA). The pellet was resuspended in 0.25 M sucrose buffer (0.25 M sucrose in buffer A) and centrifuged at 1000× *g* for 10 min.

The nuclei were counted and defined numbers (7.1 × 10^7^ nuclei/mL in 5 mL per tube) were used for PCH isolation modified from a protocol from Prusov and Zatsepina [42,43]. First, the nuclei were resuspended in 20 mL buffer B (50 mM triethanolamine–HCl (pH 7.6), 5 mM MgCl_2_, 0.2% Triton X-100 (#3051.2, Carl Roth), incubated for 5 min on ice and centrifuged at 1000× *g* for 10 min. The pellet was then resuspended in 20 mL buffer C (2 mM triethanolamine–HCl (pH 7.6), 0.5 mM MgCl_2_) and centrifuged at 1000× *g* for 10 min. The pellet was resuspended in 5 mL buffer D (2 mM triethanolamine–HCl (pH 7.6), 0.2 mM MgCl_2_) to a concentration of 7.1 × 10^7^ nuclei/mL and sonicated two times for 20 s at 20% power (250–450 Sonier, BRANSON ultrasonic corporation, Danbury, MO, USA). Each step was controlled on a light microscope. The sonicated fraction was diluted in buffer D to a volume of 10 mL, RNAseA was added to a final concentration of 1 mg/mL (#10109169001, Roche Life science products, Basel, Switzerland), and the samples were incubated at 4 °C overnight with rotation. The suspension was layered on 5 mL of 0.5 M sucrose in buffer D and centrifuged at 400× *g* for 10 min.

Then, the supernatant was layered on 5 mL 1 M sucrose in buffer D and centrifuged at 2500× *g* (without deceleration) to sediment nucleoli. Then, the supernatant was transferred to a centrifugation tube for the SW28 rotor, layered on 5 mL 1 M sucrose in buffer D, and overlaid with 15 mL buffer D for centrifugation at 27,000× *g* for 25 min to obtain the crude PCH fraction. Subsequently, the PCH pellet was resuspended in 8 mL 0.5 M sucrose in buffer E (2 mM triethanolamine–HCl (pH 7.4), 0.05 mM MgCl2) to be loaded on a sucrose gradient. For the gradient, 1.8 M sucrose (E3), 1.4 M sucrose (E2), and 1 M sucrose (E1) in buffer E were layered on top of each other in an SW28 centrifugation tube using 8 mL each, the sample was loaded on top, and the tube was filled up with buffer E. Centrifugation at 32,000× *g* for 40 min without deceleration resulted in two PCH bands between the different sucrose concentrations. The PCH bands were extracted, diluted in buffer E in a new centrifugation tube, and centrifuged at 82,000× *g* for 25 min. The resulting PCH pellet was diluted in buffer E and stored at −80 °C.

### 2.3. Quantitative Mass Spectrometry

The proteins from nuclei and PCH fractions were precipitated using methanol–chloroform precipitation (adapted from [44]). The pellet containing the proteins was dried and subsequently resuspended in denaturation buffer (6 M urea (#1.08488.1000, Merck Millipore GmbH, Darmstadt, Germany), 2 M thiourea (#1079790250, Merck Millipore GmbH), 10 mM HEPES-KOH pH 8 (#5310-100GM, Merck Millipore GmbH)) by sonication. An amount of 50 μg of each sample was reduced using 1 mM tris(2-carboxyethyl) phosphine (TCEP) (#740966.50, Macherey-Nagel, Düren, Germany) and carbamidomethylated using 5.5 mM chloroacetamide (#C0267, Merck Millipore GmbH). The digestion was performed with 0.5 μg sequencing grade endopeptidase Lys-C (Wako) for 3 h at room temperature, followed by dilution with four volumes of 50 mM ammonium bicarbonate buffer and overnight incubation with 1 μg sequencing grade trypsin (#V5111, Promega Corporation, Madison, WI, USA). The reaction was stopped by adding trifluoroacetic acid (TFA) to a final concentration of 1% and the peptides were purified on C_18_ stage tips. For dimethyl labeling, 1/4 of each sample was pooled for medium–heavy labeling while 3/4 of each sample was individually labeled with a light labeling reagent. The peptides were dried using a vacuum concentrator, reconstituted in 100 mM triethylammonium bicarbonate (TEAB) and dimethyl labeled in an automatic setup (see [45,46]). Then, each light sample was pooled with 1/4 of the medium–heavy labeled master mix and desalted using C_18_ stage tips [47]. 

The samples were measured on a Q Exactive Plus Orbitrap mass spectrometer (Thermo Fisher Scientific, Waltham, MA, USA) connected to a Proxeon EASY-nLC system (Thermo Fisher Scientific) using a high-performance liquid chromatography–tandem mass spectrometry (HPLC–MS/MS) method with data-dependent acquisition selecting the top 10 peaks for high-energy collisional dissociation (HCD) fragmentation. A volume of 5 μL sample was injected, loaded on a nano-LC column (0.074 mm × 250 mm, 3 μm Reprosil C18, Dr Maisch GmbH, Ammerbuch, Germany), and eluted using a 4 h gradient from 4% to 76% acetonitrile (solvent A: 5% acetonitrile (#10061044, Fisher Scientific GmbH, Schwerte, Germany), 0.1% formic acid (#10797488, Fisher Scientific GmbH); solvent B: 80% acetonitrile, 0.1% formic acid).

### 2.4. Mass Spectrometry Data Analysis

The database search for protein identification was performed using MaxQuant software version 1.6.5.0 [48,49] and the UniProt database [50] for Mus musculus (02/2019) without matching between runs. Carbamidomethylation was set as a fixed modification, and methionine oxidation and lysine acetylation on the protein N-terminus were set as variable modifications. The multiplicity for quantification was set to two and a false discovery rate (FDR) of 0.01 was applied. The MaxQuant output protein groups table was filtered removing contaminants and reverse hits. From the obtained protein groups table, we considered only peptides with heavy/light ratio count of two or higher. The ratios were normalized to the most frequent value of the ratios according to Geiger et al. [51]. Only the proteins reliably identified in all three replicates were selected for analysis and further filtered as described in the results. All calculations and plots were generated using the R software package (R 4.3.2) [52].

The gene ontology analysis was performed using the Gene Ontology enrichment analysis and visualization tool (GOrilla, last database update in March 2021) [53]. The genes of interest were added as the unranked target list, the list of all identified genes was added as the background list and Mus musculus was selected as the organism of the input gene list. Gene ontology terms with a *p*-value lower than 5 × 10^−5^ were considered, and the terms were grouped according to the common gene ontology term within the diagram of the GOrilla output. Redundant gene ontology terms were removed manually.

### 2.5. Amplification of Repetitive DNA from Isolated Fractions

The reaction mixture for detection of repetitive DNA in nuclei and PCH fractions by PCR contained 76 µL ddH_2_O, 10 µL PCR buffer, 1 µg DNA (brain/liver nuclei or PCH DNA), 10 µL dNTPs (2 mM each) (#K035.1 (dATP), #K037.1 (dGTP), #K038.1 (dCTP), #K036.1 (dTTP), Carl Roth), 1 µL of 10 µM forward primer, 1 µL of 10 µM reverse primer and 1 µL Taq-polymerase [54,55]. Primers are listed in Table S1 and the PCR was performed with the following steps: primary denaturation for 10 min at 95 °C, followed by 20 cycles comprising denaturation at 94 °C for 1 min, annealing at 56 °C for 1 min, elongation at 72 °C for 5 min and a final elongation step for 5 min at 72 °C. DNA of amplified products was detected by ethidium bromide staining (Sigma-Aldrich Chemie E1510) on a 1% agarose gel.

### 2.6. Slot Blot Analysis

Genomic DNA of isolated fractions was purified with Qiaex II (Qiagen, Hilden, Germany) according to the manufacturer’s protocol and concentrations were measured with a Tecan plate reader (Tecan Infinite M200, Tecan, Männedorf, Switzerland). The nitrocellulose membrane (#RPN3032D, VWR, Radnor, PA, USA) was equilibrated with SSC (10×). The genomic DNA was diluted in 6× SSC for a final concentration of 1 µg/50 µL per lane and loaded on the blot. The DNA was detected with methylene blue (#A514.2, Carl Roth), anti-methylcytosine (Table S2), or hybridized with major/minor satellite DNA probes. Probes were prepared as described under fluorescence in situ hybridization.

### 2.7. Western Blot Analysis

Mouse brain and liver nuclei, as well as C2C12 mouse myoblast cells (Table S3), were lysed in lysis buffer (0.025 M Tris HCl (pH 8) (#T1503-1, Sigma-Aldrich Chemie GmbH (Merck)), 1 M NaCl (#3957.1, Carl Roth), 0.05 M glucose (G-5400, Sigma-Aldrich Chemie GmbH (Merck)), 0.01 M EDTA (#131026.1211, AppliChem GmbH, Darmstadt, Germany), 0.2% Tween 20 (#9127.1, Carl Roth), 0.2% Nonidet P40 Substitute (#74385, Sigma-Aldrich) supplemented with protease inhibitors (1 mM phenylmethylsulfonyl fluoride (PMSF) (#6367.1, Carl Roth), 10 μM E64 (#E3132, Sigma-Aldrich), 1 μM pepstatin A (#P5318, Sigma-Aldrich)) and mechanically disrupted. The protein concentration was determined by Pierce assay (PierceTM 660 nm Protein-Assay-Kit, #22662, Thermo Fisher Scientific) using a plate reader (Infinite M200 PRO, Tecan, Männedorf, Switzerland). Subsequently, all samples were diluted in Laemmli buffer (2% SDS (#2326.2, Carl Roth), 50 mM Tris (pH 6.8), 10% glycerol (#0798.3, Carl Roth), 0.01% bromophenol blue (#A512.1, Carl Roth), 100 mM DTT), and incubated at 95 °C for 10 min. Amounts of 30 μg of each sample were analyzed by SDS-PAGE and transferred to a nitrocellulose membrane using a semi-dry blotting system at 25 V for 35–90 min. The membranes were stained with Ponceau S solution (#P7170, Sigma-Aldrich) to check for successful transfer. Then, they were blocked with 5% low-fat milk in PBS for 30 min and incubated with primary antibodies (antibodies and dilutions are listed in Table S2) in 3% low-fat milk in PBS overnight. The membranes were washed three times with 0.1% PBST (0.1% Tween 20 in PBS), incubated with secondary antibodies in 3% low-fat milk for 1 h, and washed again three times with PBST. The membranes were covered with ECL solution (Clarity Western ECL substrate, #1705061, Bio-Rad, Hercules, CA, USA) and the chemiluminescence signal was detected using an Amersham AI600 imager (Table S4).

### 2.8. Electron Microscopy and Image Analysis

Transmission electron microscopy was used to visualize the ultrastructure of nuclei and PCH fractions, either unlabeled or with immuno-labeled DNA and DNA-binding proteins. Isolated nuclei or isolated PCH were resuspended and fixed in 4% paraformaldehyde mixture. After centrifugation (500× *g* for 10 min at room temperature) the pellets of fixed material were pre-embedded in 2% low-viscosity agarose (#A9045-5G, Sigma-Aldrich Chemie GmbH (Merck)). All samples were dehydrated in ethanol at room temperature, embedded in LRWhite resin, and polymerized at 60 °C for 24 h. Thin sections made on Leica Ultracut UCT ultramicrotome were mounted on formvar/carbon-coated nickel grids. Samples were immuno-labeled with mouse anti-DNA antibody (Progen, Heidelberg, Germany) or rabbit polyclonal histone H3 tri methyl K9 (H3K9me3) antibody (Abcam, Cambridge, UK). Goat anti-mouse or goat anti-rabbit antibodies coupled with 10 nm colloidal gold (Aurion, Wageningen, Netherlands) were used as secondary antibodies. The grids with sections were pretreated with 10% normal goat serum in PBS for 10 min and then incubated for 17 h at 4 °C with primary antibody diluted in PBS containing 0.05% Tween 20 (Sigma) and 1% BSA (Fluka, Buchs, Switzerland). After washing with PBS-Tween, PBS, and 10 min goat serum treatment, sections were incubated with secondary antibodies in PBS at RT for 30 min. Grids were thoroughly rinsed with PBS, ultrapure water, and air-dried. The preparations were stained by lead citrate and uranyl acetate or by regressive EDTA technique preferential for RNP-containing nuclear structural domains or for specific DNA staining with an osmium ammine mixture at 0.2% for 1 h after HCl hydrolysis to specifically visualize DNA. The grids were examined at 80 kV in FEI Morgagni TEM equipped with a CCD camera MegaView III (Table S4). 

Images of colloid gold-stained electron microscopy samples were analyzed in ImageJ. Briefly, the original image was inverted, masked for structures of interest (nuclei, chromocenters in nuclei, and isolated chromocenters), and individual immunogold particles within the masks were counted using the ThunderSTORM plugin [56]. Statistical analysis was performed using two-tailed unpaired Student’s *t*-test. 

### 2.9. Fluorescence In Situ Hybridization

Probes for fluorescent in situ hybridization (FISH) analysis were generated by PCR. The mixture contained 69 µL ddH_2_O, 10 µL PCR buffer (100 mM Tris-HCl, 500 mM KCl (#P9541, Sigma-Aldrich Chemie GmbH (Merck)), 15 mM MgCl_2_, pH 8.3), 1 µg genomic DNA (C2C12 mouse cells) isolated via QiaexII, 10 µL dNTPs (2 mM each of dATP, dCTP, dGTP), 7 µL biotin dUTPs (1 mM) (#NU-803-BIO16-S, Jena Bioscience, Jena, Germany), 1 µL forward primer, 1 µL reverse primer (Table S1), and 1 µL Taq-polymerase. Reaction was determined to initial denaturation at 98 °C for 10 min, followed by 35 cycles of 98 °C for 1 min, 56 °C for 1 min, 72 °C for 2 min, and a final elongation at 72 °C for 5 min. PCR products were digested with DNase (Sigma 2000 u/mL, 1:500) at RT for 30 min and afterward frozen at −20 °C. FISH probes were purified by mixing 100 µL FISH probe with 25 µL sodium acetate (3 M) (#59187, Merck Millipore GmbH) and 250 µL absolute ethanol (ice cold), incubated for 1 h at −80 °C and pelleted by centrifugation at 16,000× *g* (Biofuge Fresco, Heraeus, Hanau, Germany), 4 °C for 45 min. Supernatant was removed, pellet was supplemented with 1 mL 70% ethanol (ice cold) and pelleted again by centrifugation at 16,000× *g*, 4 °C for 30 min. The supernatant was removed, and the DNA pellet was air-dried at RT for 1 h. FISH probes were resolved in 250 µL hybridization solution (50% deionized formamide (#D4551-250ML, Sigma-Aldrich Chemie GmbH (Merck)), 10% 20× SSC, 20% water, 20% dextran sulfate (50%), pH 7) by shaking at 300 rpm and 37 °C for 1 h, denatured at 98 °C for 5 min and placed on ice. 

Simultaneous to FISH probe preparation, tissue slices were rehydrated sequentially for 5 min each in the following solutions: three times in xylol (#1040, Sigma-Aldrich Chemie GmbH (Merck)), once in absolute ethanol, 96% ethanol, 90% ethanol, 80% ethanol, 70% ethanol, and three times in H_2_O. Thereafter slices were treated with sodium citrate buffer (10 mM sodium citrate, pH 6) in the autoclave (ELV 3850, Tuttnauer, Beit Shemesh, Israel) at 100 °C, 1 bar overpressure for 30 min and finally washed with H_2_O. The FISH probe was added after an equilibration period of the samples in hybridization solution at RT for 30 min. Samples were denatured at 90 °C for 5 min and hybridized at 37 °C overnight. Slices were washed with 2× SSC and PBS/0.1% Tween for 5 min, three times each. Biotin dUTP detection was performed with streptavidin-488 (#S11223, Molecular Probes, Eugene, OR, USA, 1:500) diluted in 1% BSA/PBS at RT for 1 h. Finally, slices were washed three times with PBS/0.1% Tween and PBS for 5 min. DNA was counterstained with DAPI (#6335.1, Carl Roth), washed with PBS and ddH_2_O, and mounted in Mowiol (#17951, Polysciences, Warrington, PA, USA).

### 2.10. Immunofluorescence Staining of Tissues 

Samples taken in each step of the nuclei and PCH isolation procedure were fixed in solution using 3.7% formaldehyde (#F8775, Sigma-Aldrich Chemie GmbH (Merck)). Subsequently, a few drops of each sample were transferred to microscopy slides and dried at 80 °C. For demasking, they were incubated for 3–5 min in 100 °C sodium citrate buffer (10 mM sodium citrate (#3580.1, Carl Roth), pH 6), washed with PBS, stained with 1 μg/mL 4’,6-diamidino-2-phenylindole (DAPI) for 10 min in the dark, washed and mounted with Mowiol 4-88 (#81381, Sigma-Aldrich; 4.3 M Mowiol 4-88 in 0.2 M Tris-HCl pH 8.5 with 30% glycerol) supplemented with 2.5% DABCO antifade (1,4-diazabicyclo[2.2.2]octan, #D27802, Sigma-Aldrich).

For immunofluorescence staining, the tissue slices were incubated at 60 °C for 2 h to melt the paraffin. Subsequently, they were incubated three times for 5 min in xylol for paraffin removal. Then, the tissue slices were rehydrated by sequential incubation for 5 min each in 96% ethanol, 90% ethanol, 80% ethanol, 70% ethanol, and three times in water. Antigen demasking was performed by treating the tissue slices with sodium citrate buffer at 100 °C for 30 min in an autoclave. The tissue slices were equilibrated for 15 min in PBS, permeabilized with 0.7% Triton X-100 (#T8787, Sigma-Aldrich Chemie GmbH (Merck)) in PBS two times for 15 min and washed three times with PBS. The tissue slices were circled with a liquid-blocking pen and blocked with 4% BSA (#2834.3, Carl Roth) for 30 min. Incubation with primary antibodies (Table S2) was performed overnight at 4 °C, followed by three times washing with PBST (0.1% Tween 20 in PBS) for 10 min and secondary antibody (Table S2) incubation for 1 h at room temperature. The tissue slices were washed three times with PBST for 10 min, counterstained with 1 μg/mL DAPI, washed with PBS and water, and mounted using Mowiol as described above.

### 2.11. Cell Culture and Transfection

C2C12 mouse myoblast cells (see Table S3) were grown in Dulbecco’s Modified Eagle Medium (DMEM) with high glucose (#D6429, Sigma-Aldrich) supplemented with 20% fetal bovine serum (#F7524, Sigma-Aldrich), 1× glutamine (#G7513, Sigma-Aldrich), and 1 μM gentamicin (#G1397, Sigma-Aldrich) at 37 °C and 5% CO2 in a humidified incubator. Tests to check for potential mycoplasma contamination were performed regularly.

The C2C12 cells were cotransfected with plasmids expressing histone GFP-H1 [57] and MeCP2-Halo (see Table S5) at different ratios (2:1, 1:1, 1:2) using the Neon transfection System (Thermo Fisher Scientific) according to the manufacturer’s instructions. Voltage, width, and pulse for C2C12 cells were: 1650 V, 10 ms, and 3 pulses. Then, cells were seeded onto coverslips and grown at 37 °C and 5% CO_2_ in a humidified incubator. Twenty-four hours after transfection, Halo Tag R110 Direct Ligand (G 3221, Promega GmbH, Walldorf, Germany) was added to the medium to visualize MeCP2.

### 2.12. Immunofluorescence Staining of Cells

At 48 h after transfection, cells were washed with PBS and fixed with 3.7% formaldehyde for 12 min. After fixation, cells were permeabilized with 0.5% Triton X-100 in PBS for 10 min and washed three times with PBST (0.02% tween 20 in PBS). Then cells were blocked with 2% BSA in PBST for 30 min and incubated with primary antibody to histone H1 (Table S2) for 1 h. After washing three times with PBST, cells were incubated with secondary antibody against mouse IgG conjugated with Cy3 (715-166-151, Jackson ImmunoResearch Europe Ltd., Cambridgeshire, UK) (Table S2) for 1 h in the dark, followed by three times washing with PBST, 8 min DAPI staining in the dark, washing with PBST for two times and water once. Finally, cells were mounted in Mowiol 4-88 (#81381, Sigma-Aldrich; 4.3 M Mowiol 4-88 in 0.2 M Tris-HCl pH 8.5 with 30% glycerol) supplemented with 2.5% DABCO antifade (1,4-diazabicyclo (2.2.2)octan, #D27802, Sigma-Aldrich) and stored at −20 °C till imaging.

### 2.13. Fluorescence Microscopy and Image Analysis

To analyze the distribution of major satellite and minor satellite DNA in tissues, confocal microscopy imaging was carried out on an UltraView VoX spinning disk microscopy system with a 60× objective and 0.3 µm intervals for z-stacks (see Table S4). The single nuclei were segmented based on the DAPI channel using the Volocity software (v. 6.3., Perkin Elmer, Waltham, MA, USA). The FISH probe signal was applied to segment the major and minor satellite foci. The number of foci per nuclei and individual foci size were measured. The total foci volume was calculated by the sum foci volume per nuclei and plotted by the R software package [52].

To evaluate the PCH isolation procedure, nuclear fractions at different steps were DAPI stained and visualized on a Zeiss Axiovert 200 or a Zeiss Axioplan microscope with a 40× objective (see Table S4). The images were processed using ImageJ/FIJI software (v. 1.54) [58,59].

To analyze the distribution of selected proteins based on the mass spectrometry, immunostained tissue slides were imaged on an UltraView VoX spinning disk microscopy system with a 60× objective and 0.3 µm intervals for z-stacks as described above. The nuclei and PCH were segmented based on the DAPI channel using Volocity software (Perkin Elmer). The PCH number per nucleus and the individual PCH volumes were measured. The heterochromatin accumulation was calculated as the ratio of the mean PCH intensity per nucleus versus the nucleoplasm intensity. The violin plots and the significance tests were created using the R software package [52].

To evaluate the possible interplay between MeCP2 and histone H1, immunofluorescence-stained C2C12 cells were imaged using Nikon Crest microscope with a 60× objective (see Table S4). The nuclei and PCH were segmented based on the DAPI channel using ImageJ/FIJI software [58,59]. The nuclei size and mean fluorescence intensities of DAPI, MeCP2, and H1 in nuclei, the individual PCH number, size, and mean fluorescence intensities within nuclei were measured. The sum fluorescence signals per nuclei were calculated by mean fluorescence intensities × nuclei size. The sum fluorescence signals in PCH per nuclei were calculated by the accumulation of sum fluorescence in each PCH (mean fluorescence × size of PCH). The mean fluorescence intensities in PCH per nuclei were calculated by ratio of sum fluorescence signals of all PCH per nuclei to sum PCH size of the nuclei. The fold enrichment of fluorescence in PCH per nuclei (heterochromatin accumulation) was calculated by the ratio of mean fluorescence in PCH per nuclei to the mean fluorescence in nucleoplasm. The fraction of fluorescence located in PCH per nuclei was calculated by the percent ratio of total fluorescence in PCH per nuclei to total fluorescence in the nuclei. The final plots were generated using the R software package [52].

All example images were processed using ImageJ/FIJI software [58,59].

## 3. Results

### 3.1. (Peri)Centromeric Heterochromatin Organization in Mouse Organs

**Figure 1:**
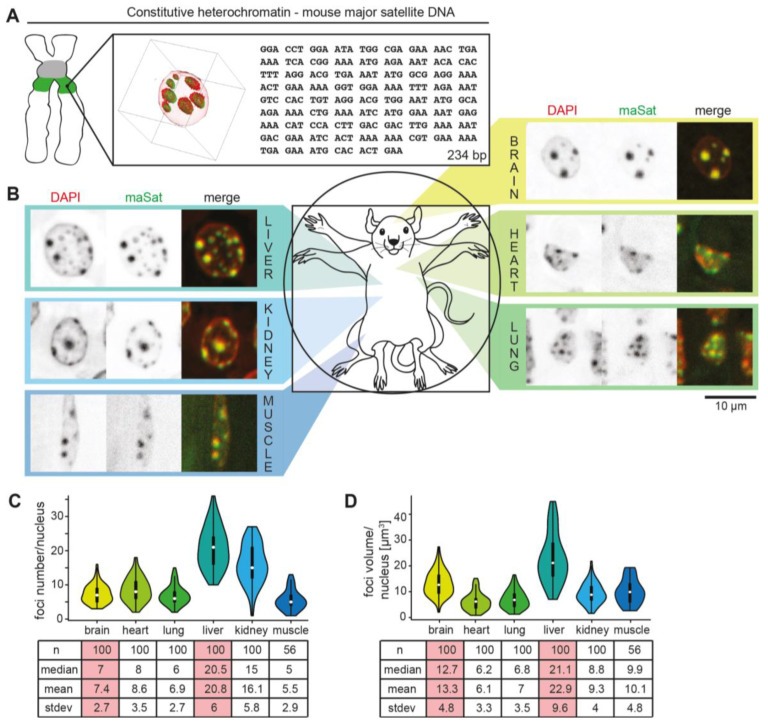
Pericentromeric heterochromatin organization in mouse tissues. (**A**) Rotated view of 3D projection of major satellite (pericentromeric) DNA fluorescence in situ hybridization (FISH; green) overlaid with the DNA stained with DAPI (red) and major satellite DNA repeat sequences. (**B**) Major satellite (maSat) DNA FISH (green) overlaid with DNA (red) in different mouse tissues as indicated. (**C**,**D**) Quantitation of major satellite foci in different mouse organs. Violin plots represent the median foci number (white mark), spread (line; upper and lower quartile), the whiskers the 95% interval, and the rotated kernel density plot all possible values. Corresponding statistics for major satellite foci number (**C**) and volume (**D**) per nucleus calculated by Volocity software after segmenting single foci.

To identify and analyze the distribution of (peri)centromeric DNA (Figure 1A and Figure S1A) in vivo, different mouse tissues were hybridized with probes specific for the pericentromeric major satellite and the centromeric minor satellite DNA repeats. In all tissues tested, major satellite signals clearly colocalized with DNA-dense regions of the mouse nuclei corresponding to pericentromeric heterochromatin (PCH) clusters (Figure 1B). The minor satellite DNA was clustered next to the PCH regions (Figure S1B). Further, brain, heart, lung, and muscle nuclei revealed fewer but larger PCH whereas the kidney and liver showed more but smaller PCH (Figure 1B). Moreover, the minor satellite foci distribution follows the PCH distribution in different tissues (Figure S1B). The quantification analysis of the major and minor foci number and size per nucleus confirmed these observations (Figure 1C,D and Figure S1C). As PCH are hallmarked with high DNA and H3K9me3 density, the mouse brain and liver sections were immunostained with anti-DNA or anti-H3K9me3 antibodies and imaged using transmission electron microscopy (Figure S2). The densities of Au particles were measured and calculated in the selected PCH region (PCH), in all PCH of selected nuclei (PCH in nu), and in the whole nuclei (nu) (Figure S2). Both the brain and liver showed high signal densities in the PCH regions and lower average signals in the whole nuclei due to low signals outside PCH (in the euchromatin and nucleoplasm).

Thus, although constitutively silenced in all tissues, the PCH is actually dynamically regulated in a tissue-specific manner. We hypothesized that proteomic changes underlie the biophysical changes and decided to investigate further the proteomic similarities and differences in the mouse brain and liver PCH as they show characteristic differences in PCH compartment size and number.

### 3.2. Isolation of the Native PCH Fraction from Mouse Tissues

**Figure 2:**
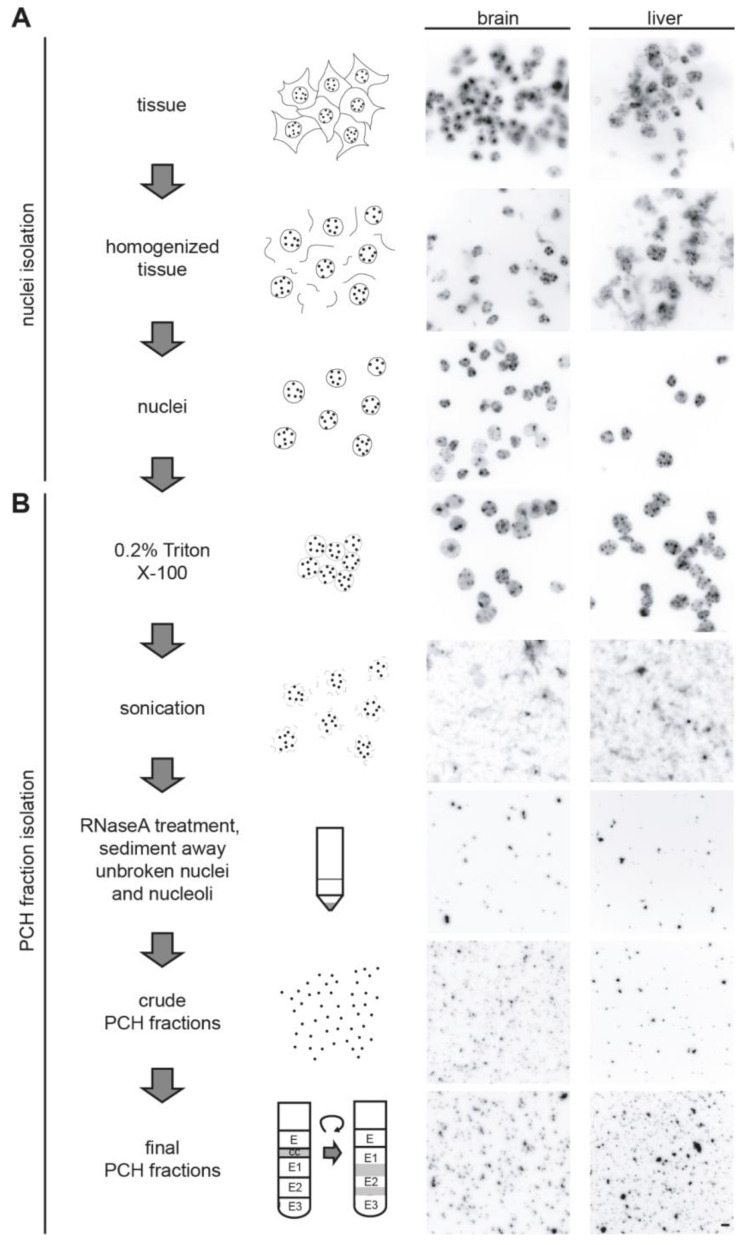
Nuclei and PCH isolation from mouse brain and liver tissues. Scheme of the workflow for isolation of nuclei (**A**) and PCH fraction (**B**) from mouse brain and liver tissues with the representative images of DNA (DAPI) staining at each step. Scale bar 5 μm.

To analyze the protein compositions of the PCH fractions by mass spectrometry, we first isolated PCH fractions from the mouse brain and liver in view of their distinct PCH organization. Therefore, we adapted and modified a protocol from Prusov and Zatsepina [42,43]. In comparison to previously used enzyme/salt/affinity-based methods for heterochromatin isolation [60,61,62,63], this protocol is based on a series of sucrose gradient centrifugation steps, and thus unbiased, albeit at the cost of some possible contaminants from other subnuclear fractions. The individual steps and representative DAPI-stained images of the protocol are depicted in Figure 2.

### 3.3. Validation of the PCH Isolation Strategy

**Figure 3:**
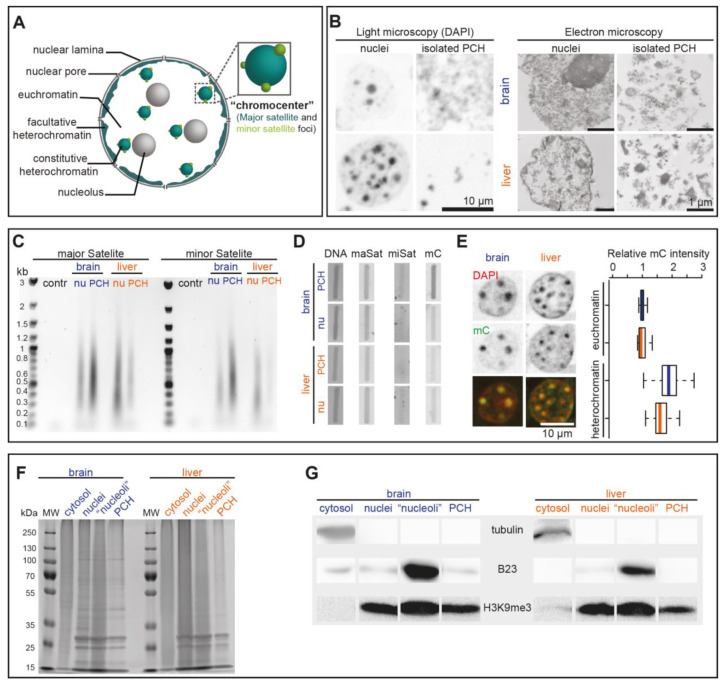
Characterization of purified nuclei and PCH fractions at DNA and protein level. (**A**) Scheme of interphase cell subnuclear compartments with an integrated comparison of minor and major satellite distribution. (**B**) Side-by-side comparison of light and electron microscopy of DNA distribution in nuclei and isolated PCH fractions from brain and liver as indicated. (**C**) Ethidium bromide-stained agarose gel electrophoresis analysis of PCR-amplified major and minor satellite repeats from nuclei (nu) and PCH fractions (control PCR (contr) without DNA) as indicated. (**D**) Slot blot analysis of DNA isolated from nuclei and PCH fractions from brain and liver as indicated. Columns show DNA stained with methylene blue as loading control (left), hybridization with minor and major satellite probes as indicated (middle), and antibody detection of methylcytosine (mC) (right). (**E**) Quantitative immunofluorescence analysis of methylcytosine (mC) in euchromatin versus heterochromatin in brain and liver tissue sections with corresponding statistics. Boxplots represent the median mC intensity (line) normalized to brain euchromatin, spread (box; upper and lower quartile), and the whiskers at the 95% interval. The statistics are summarized in Table S6. (**F**,**G**) Coomassie-stained SDS-PAGE analysis (**F**) and Western blot analysis (**G**) of purified fractions as indicated. cytosol: cytosolic fraction; “nucleoli”: assumed “nucleoli” fraction; PCH: isolated pericentric heterochromatin fractions.

In eukaryotic cells, nuclei are subdivided into different subcompartments with distinct biophysical/chemical characteristics such as PCH (major satellite foci) and minor satellite foci (Figure 3A). The minor satellite foci are located adjacent to the PCH compartments as shown in Figure 1B and Figure S1B. As the PCH colocalized with highly condensed and DAPI-densely stained chromatin regions, we investigated and compared the isolated nuclei and PCH via light microscopy and transmission electron microscopy (Figure 3B). Similar to the DNA FISH results for major satellites (Figure 1B), the isolated brain nuclei exhibited fewer but larger densely DAPI-stained PCH compartments than liver nuclei (Figure 3B). Moreover, the isolated PCH fraction from both the brain and liver could be detected with high compaction (dense signals) but with possible contaminants (light signals) (Figure 3B). Alternatively, the final PCH fractions became partially less condensed, leading to differential signal intensities (Figure 3B).

To check the purity of isolated PCH fractions, the DNA sequences were isolated from purified nuclei and PCH fractions and used as templates for PCR using major and minor satellite DNA-specific primer pairs. Both major and minor satellite DNA were detected in isolated brain and liver PCH fractions (Figure 3C), indicating that minor satellites were included in the PCH fraction. In parallel, the same amount of isolated DNA was directly transferred to a membrane (Figure 3D left) for DNA–DNA hybridization using major or minor satellite DNA probes (Figure 3D middle two lanes) and for slot blot with anti-mC antibody (Figure 3D right). The major satellite DNA was detected in all samples used, while minor satellite DNA was rarely detected due to low loading amount and abundance. Further, the isolated brain PCH showed significantly higher mC levels in isolated brain PCH than in other fractions, which was further confirmed by immunostaining against mC in mouse brain and liver sections (Figure 3E).

To check the purity of isolated PCH at the protein level, the same amount of isolated cellular fractions (cytosolic fraction, nuclei, assumed “nucleoli”, and isolated PCH) from both mouse brain and liver were analyzed by SDS-PAGE followed by Coomassie staining (Figure 3F) and Western blot using cytoplasm (tubulin), nucleoli (B23), and heterochromatin (H3K9me3) markers (Figure 3G). As compared to the whole nuclei extracts, the PCH fractions showed distinct protein composition in both the brain and liver (Figure 3G). Tubulin was solely detected in the cytoplasm, confirming the proper isolation of nuclei from both the brain and liver. The B23 was mainly detected in the assumed “nucleoli” fraction from both the brain and liver and detected, albeit to a very low amount, in the isolated brain PCH, suggesting lower purity of brain PCH relative to liver PCH fractions. H3K9me3 was detected in all fractions except the cytoplasm, confirming that the isolated PCH fractions actually enrich the major satellite chromatin and that the assumed “nucleoli” contain both nucleoli and heterochromatin fractions.

In conclusion, the protocol was successfully used to specifically enrich the PCH fractions hallmarked with major satellite DNA, high DNA density, and high mC and H3K9me3, although with other contaminants to some extent.

### 3.4. Quantitative Proteomic Analysis of PCH-Enriched Proteins

**Figure 4:**
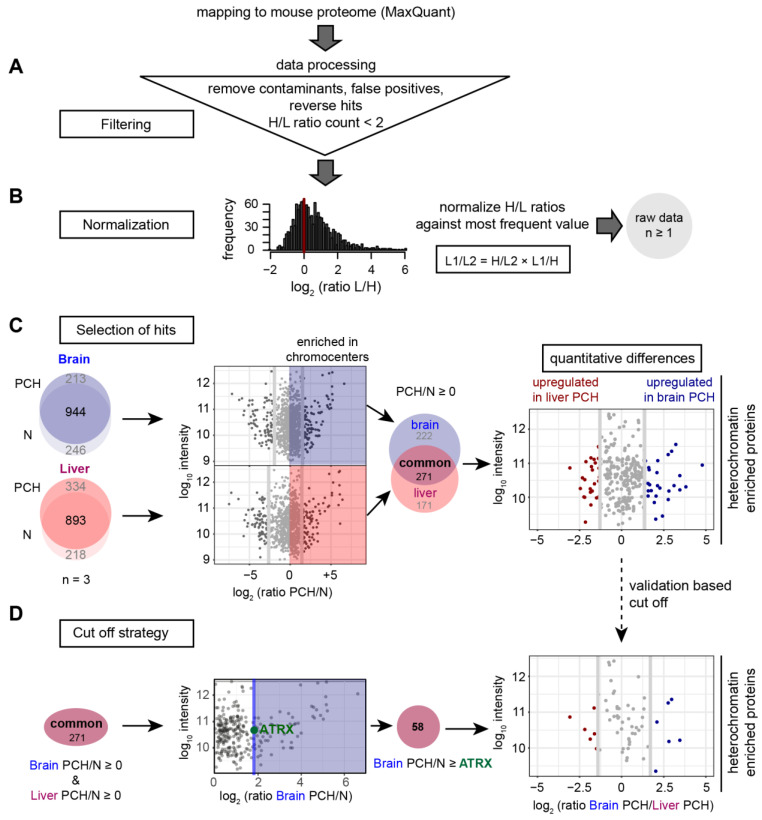
Workflow for the quantitative mass spectrometry analysis. The data were analyzed using MaxQuant software by mapping the peptides identified against the UniProt mouse proteome. (**A**) The data were filtered by removing contaminants, false positives, and reverse hits identified by MaxQuant. Proteins with a heavy/light (H/L) ratio count ≥2 were considered for further analysis. (**B**) The H/L ratios were normalized against the most frequent value and the different fractions were compared to each other by dividing one heavy/light ratio by the other (H/L1/H/L2 = H/L1 × L2/H = L2/L1). (**C**) The proteins identified in all three biological replicates (n = 3) were considered for further analysis (**left**). The log2 ratios of protein intensities identified in brain (blue) and liver (red) PCH to that in nuclei were plotted against the relative protein intensities (log10 intensity). Next, the common proteins enriched in both brain and liver PCH fractions (labeled with “common”) were filtered as PCH-enriched protein candidates (**middle**). To identify proteins that were differentially accumulated in PCH fractions from different tissues, the log10 intensity was plotted against the log2 ratios of protein intensity in brain PCH to that in liver. The upper and lower 10% of the proteins were considered up- or downregulated (more or less accumulated) in brain or liver PCH (**right**). (**D**) A validation-based cut-off was applied to the proteins from (**C**). The log10 protein intensities were plotted against the protein enrichment levels (the ratio of protein intensity in PCH to that in the nucleus). Only the proteins with higher accumulation levels than ATRX in the brain were selected as PCH-accumulated proteins (58 hits). Then the log10 intensity was again plotted against the log2 ratios of protein intensities in brain PCH to that in liver PCH, and the upper and lower 10% of the proteins were considered up- or downregulated (more or less accumulated) in brain or liver PCH.

The nuclei and PCH fractions were further processed for quantitative HPLC-MS/MS measurements. The mass spectrometry data were quantitatively analyzed as described in Figure 4 including data filtering, normalization, and hits selection (Figure 4A–C). Then, the H/L ratios were normalized to the most frequent value according to Geiger et al. [51] (Figure 4B). The resulting raw data contain all hits detected in at least one replicate from any sample (Figure 4B).

To identify proteins consistently enriched in PCH, a step-by-step selection strategy was adopted and evaluated (Figure 4C). First, we selected protein hits that were (1) reproducibly identified in all three biological replicates (n = 3) (Figure 4C, left) and (2) enriched in PCH in comparison to the nuclei (PCH/nuclei > 1) (Figure 4C, middle). Hits were identified to be enriched in the brain and liver PCH separately, including some that were consistently accumulated in both brain and liver PCH fractions. Then, to obtain the proteomic similarities/differences between brain and liver PCH quantitatively, the proteins enriched in both brain and liver PCH were selected, and the log2 values of protein intensities from brain PCH to liver PCH were plotted against the log10 intensities (Figure 4C, right and Figure S3). The upper and lower 10% of the proteins were considered up- or downregulated (more or less accumulated) in brain or liver PCH (Figure S3).

The proteins more enriched in the brain PCH comprised the chromatin proteins MeCP2, ATRX, and CHD5, as well as several transcriptional regulators (Figure S3). However, the proteins highly enriched in the liver PCH comprised the chromatin proteins DEK, RCC1, SMARCD2, and ZNF281, which play a role in transcriptional regulation, as well as the histones H2A.Z and H1.4 (Figure S3). The proteins enriched in PCH with equal abundance in the brain and liver PCH (conserved PCH proteins) comprised histones, chromatin proteins, and proteins involved in the regulation of transcription, all (or part) of which might play a general but essential role for PCH dynamics regardless of cell types. Moreover, various membrane- and RNA-associated proteins that are located in compartments abutting the PCH (nucleolus, nuclear membrane) were also identified in both PCH fractions.

Next, the gene ontology (GO) analysis of all the candidate proteins enriched in both brain and liver PCH (Figure S3) was performed using the GOrilla tool [53] (Figure S4). The GO analysis terms for biological processes revealed that many genes were associated with metabolic processes, including the regulation of gene expression and DNA-templated transcription (Figure S4). The biological processes with the highest enrichment values were nucleosome and nucleus organization, chromosome segregation, but also nuclear pore organization, protein–DNA complex disassembly, and nuclear export. For the category of molecular function, the GO terms chromatin and nucleosome binding as well as structural molecule activity, and constituent of the ribosome and of the nuclear pore. For the category of cellular components, the GO terms and highlights the cohesin complex, THO complex, nucleosome, nuclear pore, and ribosomal subunit.

In summary, many PCH proteins identified in the brain and liver are associated with typical GO terms for heterochromatin proteins (e.g., nucleic acid binding, nucleosome/chromatin binding/organization, chromatin segregation, and gene expression). Nevertheless, proteins associated with nuclear pores, the ribosome, and RNA were also identified due to the copurification of PCH adjacent nuclear components.

### 3.5. The Isolated PCH Contains Nuclear Membrane- and RNA-Related Protein Hits

The quantitative mass spectrometry analysis of isolated PCH fractions revealed several proteins associated with the nuclear membrane (e.g., nuclear pore proteins) and RNA processing (e.g., RNA synthesis and splicing). Due to the functional roles of these proteins, they are not likely to localize within PCH, and, on the contrary, they are much more likely to localize close to PCH and be, thus, co-purified during PCH isolation. Therefore, we analyzed the localization of membrane- or RNA-related proteins by immunofluorescence staining on tissue slices and subsequent imaging (Figure S5). In parallel, Western blot analysis was performed to confirm the antibody specificity and to detect the protein abundance in the mouse brain and liver nuclei (Figure S6). All of the selected nuclear membrane-related proteins were located surrounding the nuclei together with comparable abundance in both tissues (Figures S5A and S6A). For the splicing factors, both SNRNP70 and MBNL2 form specific foci (nuclear speckles [64]) outside PCH foci together with similar expression levels (Figures S5B and S6B). For the transcription-related factors, SIN3A and ZNF281 were not enriched at PCH foci in both tissues, while GATAD2b was enriched at PCH foci in liver nuclei, although GATAD2b was more abundant in the brain (Figures S5C and S6C).

In conclusion, all tested proteins with GO terms for the nuclear membrane, RNA, or transcription did not enrich or only tissue-specifically enriched (GATAD2b) at PCH compartments in the mouse brain or liver.

### 3.6. The Isolated PCH Is Enriched in Histones and Chromatin Related Proteins

In addition to the multiple nuclear membrane- and RNA-related candidate hits, large amounts of chromatin-related proteins and histone variants were identified in the PCH fraction from both tissues. Thus, we further examined if these candidates are indeed enriched in PCH compartments in vivo.

**Figure 5:**
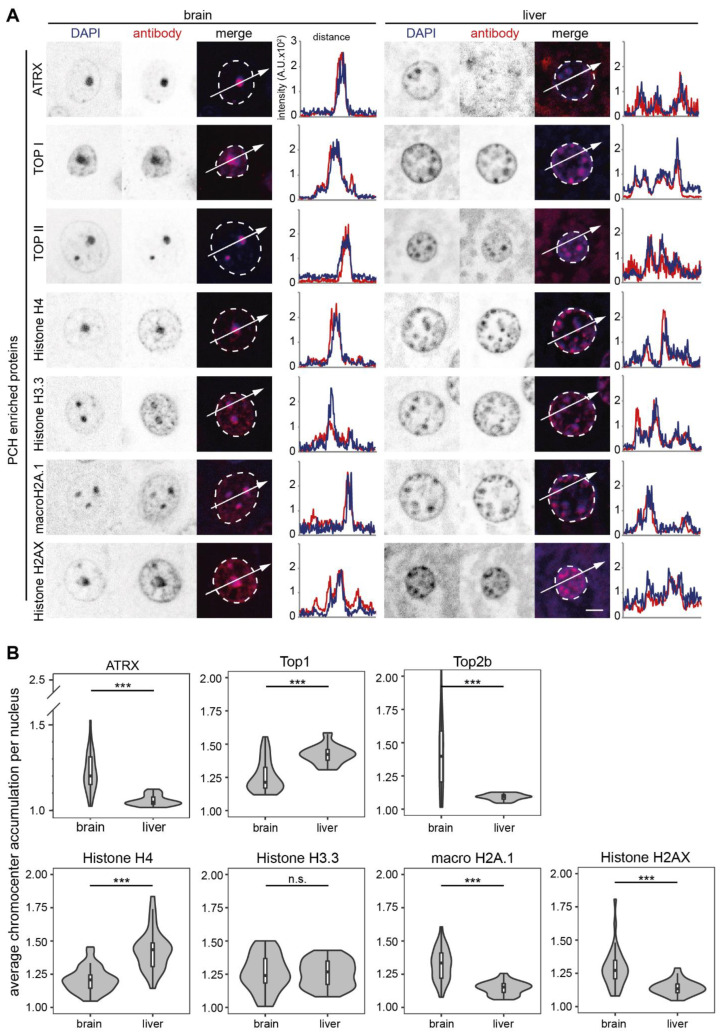
Candidates with both PCH accumulation and GO terms for chromatin in mouse brain and liver. The candidates with a high frequency of GO terms for chromatin were detected on mouse brain and liver tissue slices by immunofluorescence staining, and the candidates with consistent PCH accumulation in both mouse brain and liver tissues are shown. (**A**) Immunofluorescence staining on mouse brain and liver tissue slices. The nuclear outlines are marked in white on the merged channel image. Line plots of the fluorescence intensity in arbitrary units (A.U.) plotted against the distance depict the colocalization of the antibody staining (red) with the DNA counterstain (DAPI, blue). (**B**) Protein PCH accumulation analysis in brain and liver slices. Violin plots depict the average PCH accumulation per nucleus calculated as the ratio of PCH intensity versus nucleoplasm intensity after nucleus and PCH segmentation using Volocity software (see Figure S7). The *p*-values were calculated using the Wilcoxon rank test. *** *p* < 0.001, n.s.: not significant. The statistics are summarized in Table S6.

**Figure 6:**
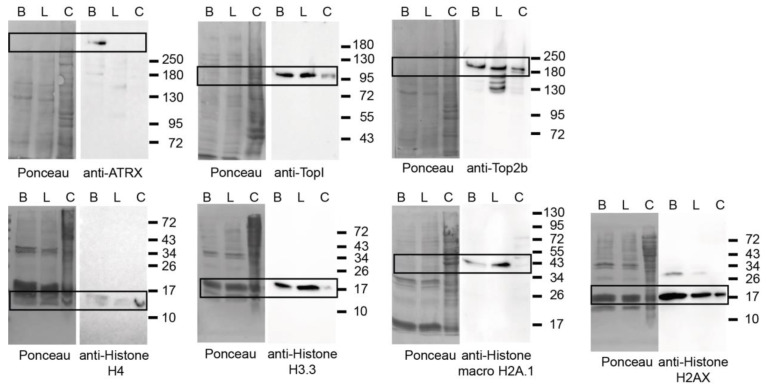
Protein level analysis of candidates with both PCH accumulation and GO terms for chromatin in mouse brain and liver. The protein levels of candidates with both PCH accumulation and GO terms for chromatin in mouse brain and liver were detected by Western blot analysis. B: brain nuclei lysate, L: liver nuclei lysate, C: whole cell lysate from mouse myoblasts. The protein transfer efficiencies are shown by Ponceau S staining on the left, and the antibody signals with chemiluminescence detection are shown on the right. The molecular weight markers indicate the protein mass in kDa and the black boxes mark the bands of interest.

Topoisomerases I and II were chosen as conserved PCH candidates, while ATRX and SMARCD2 were selected due to their higher accumulation in brain and liver PCH, respectively (Figure S3). Topoisomerases are ubiquitous proteins and capable of changing the topology of DNA via transient breaks during DNA replication, transcription, and recombination [65]. We found that topoisomerases I and II were consistently enriched at PCH together with similar total protein amounts in the whole nuclei but differential PCH accumulation levels (Figure 5A and Figure 6), in line with the reported functions of topoisomerases in heterochromatin organization [66]. However, topoisomerases I showed higher PCH accumulation in the liver while topoisomerases II showed higher PCH accumulation in the brain, indicating possible tissue-specific functions of topoisomerases I and II in the PCH region (Figure 5B and Figure S7). Although locally accumulated at PCH in both tissues, ATRX was highly expressed in brain tissue together with higher PCH accumulation (Figure 5, Figure 6 and Figure S7), suggesting the role of ATRX in PCH organization during brain development [67,68,69]. SMARCD2 did not accumulate at both brain and liver PCH foci regardless of the total protein levels (Figure S8). SMARCD2 is a member of the mating-type switching (SWI)/sucrose nonfermenting (SNF) ATP-dependent chromatin remodeling complex (SWI/SNF). The SWI/SNF was reported to mediate epigenetic and gene transcription regulation (reviewed in [70]). In addition to SMARCD2, we also identified several SWI/SNF subunits including core subunit SMARCB1 and SMARCA2 (Figure S3) [70]. Moreover, SMARCB1 and SMARCA2 were equally expressed in both tissues but were not significantly enriched in PCH except that SMARCA2 formed foci in liver nuclei, which partially overlapped with PCH (Figure S8). Thus, the SWI/SNF subunits were probably copurified due to the PCH fraction contaminants from adjacent, less compacted eu-/heterochromatin.

Histone proteins are among the most abundant proteins in eukaryotic cell nuclei as the structural basis of chromatin. Consistently, histone proteins and their variants were identified to be enriched in both brain and liver PCH with similar abundance except for histone H1.4 and H2A.Z., which were more abundant (upregulated) in liver PCH (Figure S3). Histone H4, H3.3, macroH2A.1, and H2AX showed clear PCH enrichment and comparable protein levels in both tissues except for the macroH2A.1 (Figure 5, Figure 6 and Figure S7). The macroH2A.1 was more expressed in liver nuclei (Figure 6).

In summary, hit selection with a signal ratio of PCH/nuclei > 1 identified both PCH-accumulated (e.g., ATRX) and common chromatin-bound proteins (e.g., SWI/SNF subunits), as well as nuclear membrane-, RNA-, or transcription-related proteins due to the nuclear non-PCH contamination. Thus, further filtering steps are required/necessary to specify the PCH-enriched proteins.

### 3.7. Proteomic Analysis of Heterochromatin-Enriched Proteins

The immunofluorescence stainings shown above confirmed that most identified hits related to nuclear membrane, RNA, and transcription regulation did not actually enrich at PCH. Thus, we performed a validation-based filtering of the mass spectrometry data. As ATRX showed the lowest enrichment in brain PCH among the PCH-enriched proteins and is located at the boundary of PCH-enriched and -unenriched proteins (Figure S9), we decided to remove all possible false positive hits using ATRX as the threshold (Figure 4D).

**Figure 7:**
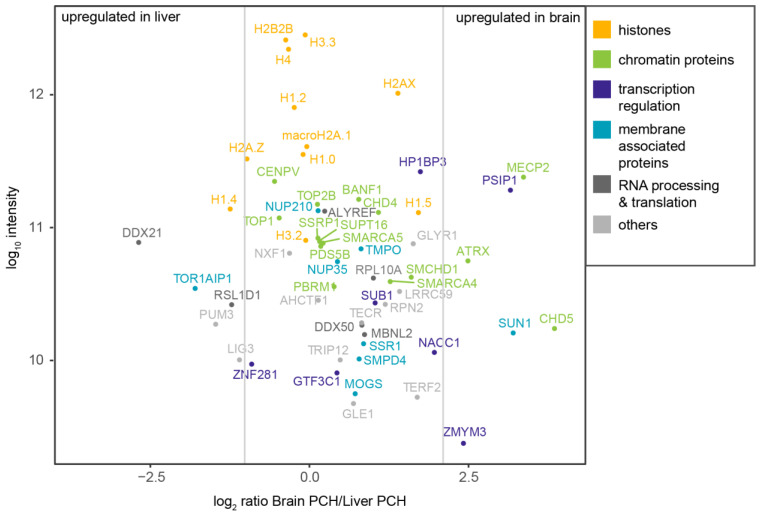
PCH-accumulated proteins identified by mass spectrometry and the validation-based cut-off. The dot plot shows the log10 intensity plotted against the log2 ratio of brain PCH intensity versus liver PCH intensity. Vertical lines indicate the three categories: proteins enriched in the liver PCH, proteins with similar abundance in the brain and liver PCH, and proteins enriched in the brain PCH. The color code indicates the protein function manually assigned based on the UniProt webpage functional information. Histones are labeled in orange, chromatin proteins in green, proteins involved in transcriptional regulation in dark blue, membrane-associated proteins in cyan, proteins involved in RNA processing or RNA-binding proteins in dark gray, and proteins not fitting into any of these categories in light gray.

Briefly, the data shown in Figure S3 were taken as raw data. Then, the ATRX accumulation levels in the brain (ratio of ATRX intensity in brain PCH to brain nuclei) were applied as a filtering boundary. Only the proteins with higher accumulation levels than ATRX in brain PCH were selected. As a result, we obtained a smaller selection of PCH-enriched proteins with possibly common or tissue-related roles in heterochromatin organization (Figure 7).

**Figure 8:**
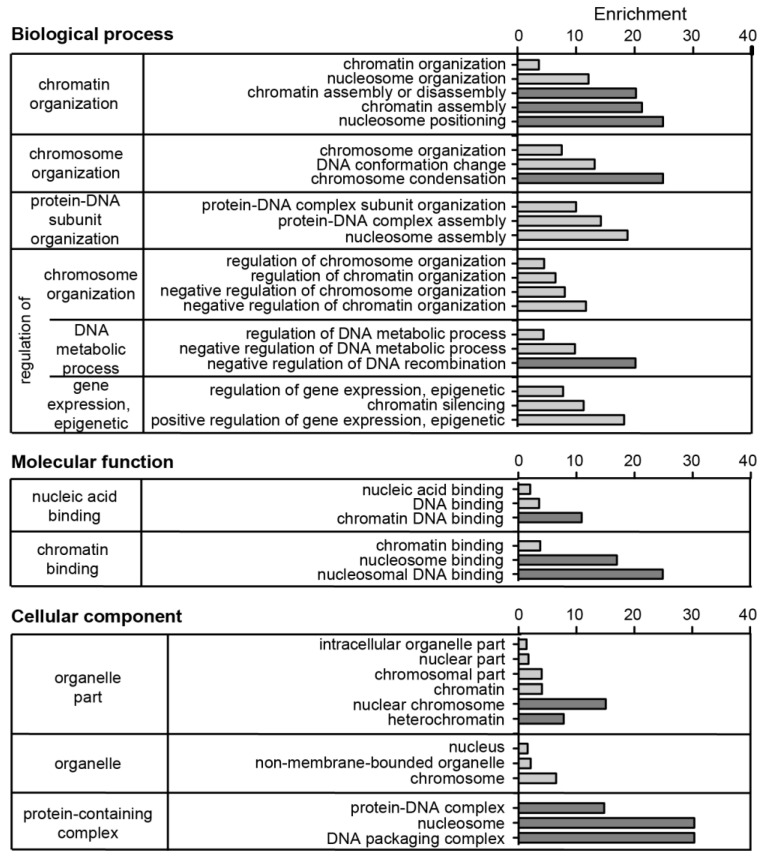
Gene ontology (GO) analysis of identified PCH accumulated proteins after validation-based cut-off. The protein list was subjected to the GOrilla tool [53] for gene ontology analysis in the categories of biological process, molecular function, and cellular component. The proteins enriched in heterochromatin were added to the target list and all identified proteins (without cut-off or filtering) as the background list. GO terms with a *p*-value lower than 5 × 10^−5^ were considered. The GO terms were grouped according to the highest common gene ontology term within the diagram of the GOrilla output, and redundant terms were removed manually. Plotted is the number of genes in the GO term (b) as % of total genes in the target list (n) and the enrichment calculated by the GOrilla tool as (b/n)/(B/N) with b: number of genes in the target list associated with specific GO term; n: total number of genes in the target list; B: number of genes in the background list associated with specific GO term; N: total number of genes in the background list. The five terms (biological function, cellular component) or three terms (molecular function) with the highest enrichment in the categories are highlighted in dark gray.

Functional enrichment analysis of the PCH-enriched proteome shows that the GO terms for biological process and molecular function are well in line with the known functions related to chromatin activities, including chromatin binding, assembly, condensation, and nucleosome positioning (Figure 8). In parallel, the GO terms for cellular components showed an enrichment for chromosome and heterochromatin (Figure 8).

In summary, all GO terms relate to protein functions involved in chromatin packaging in nucleosomes and heterochromatin fractions. Thus, the unbiased PCH isolation approach in combination with a rigorous validation-based cut-off represents a system for the reliable identification of PCH proteins.

### 3.8. MeCP2 Competes with Histone H1 for Heterochromatin Binding and Compartmentalization

**Figure 9:**
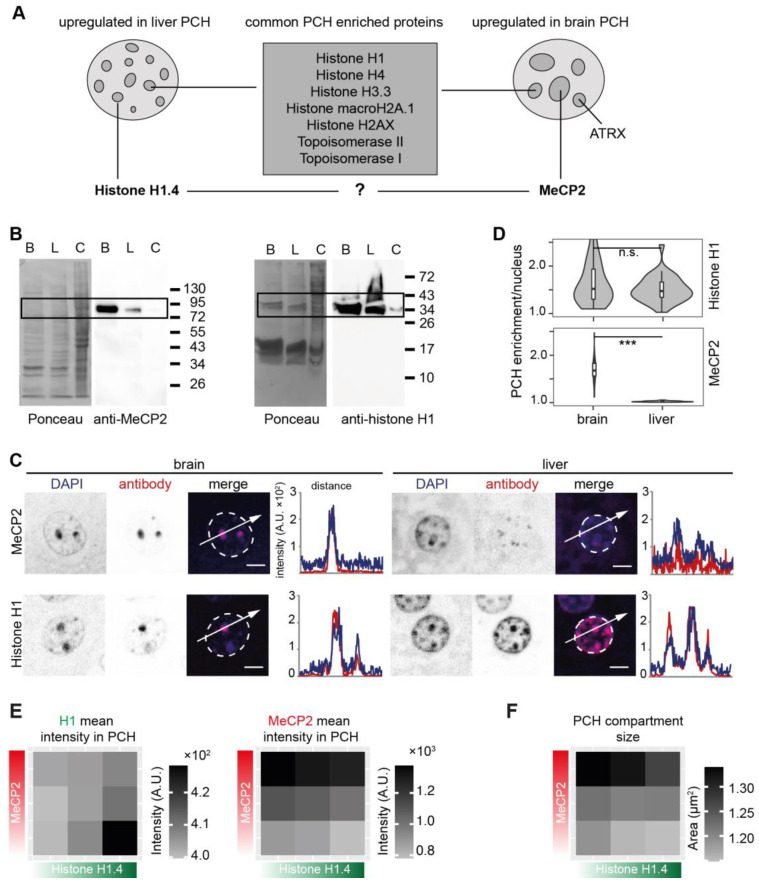
MeCP2 repulses histone H1 for PCH fusion. (**A**) Schematic summary of the PCH-accumulating proteins validated by immunofluorescence staining. The nuclei schemes mimic the differing PCH organization in mouse brain and liver nuclei. Most of the histones/histone variants and the topoisomerases I and II colocalize and accumulate at PCH foci to a similar extent in both brain and liver tissues. ATRX is more accumulated in brain PCH. Interestingly, histone H1.4 and MeCP2 are differentially accumulated in brain and liver tissues. (**B**–**D**) Comparison of MeCP2 and histone H1 in mouse brain and liver. (**B**) Western blot analysis to detect the protein levels of MeCP2 and histone H1 in brain and liver nuclei. B: brain nuclei lysate; L: liver nuclei lysate; C: whole mouse myoblast cell lysate. (**C**) Immunofluorescence staining on mouse brain and liver tissue slices using antibodies against MeCP2 and histone H1, separately. The nuclear outlines are marked in white on the merged channel image. Line plots of the fluorescence intensity in arbitrary units (A.U.) plotted against the distance depict the colocalization of MeCP2 or H1 (red) with the DNA counterstain (DAPI, blue). Scale bar 5 μm. (**D**) PCH accumulation analysis of MeCP2 and histone H1 in brain and liver slices. Violin plots depict the average PCH accumulation per nucleus calculated as the ratio of PCH intensity versus nucleoplasm intensity after nucleus and PCH segmentation using Volocity software (see Figure S7). The *p*-values were calculated using the Wilcoxon rank test. *** *p* < 0.001, n.s.: not significant. The statistics are summarized in Table S6. (**E**,**F**) The interplay between MeCP2 and histone H1.4 in cultured C2C12 cells with ectopic overexpression strategy. The cells were cotransfected with plasmids expressing MeCP2-halo and GFP-histone H1.4 and stained with anti-halo antibody (Table S2). Images were taken using a Nikon crest microscope and analyzed using FIJI as described in Figure S10. (**E**) The mean intensities of MeCP2 and H1.4 in PCH/nuclei were calculated as the ratio of the sum intensities in all PHC/nuclei to sum PCH area/nuclei and plotted using Excel. The statistics are summarized in Table S7. (**F**) The mean PCH compartment size was obtained by dividing the total PCH area/nuclei by the PCH number. Color scale indicates the mean values. The statistics are summarized in Table S7.

Further, the cut-off strategy was applied to identify possible hits that are differentially accumulated at mouse brain and liver PCH. Linker histone H1 variant H1.4 was classified as the protein candidate that was more PCH-accumulated (upregulated) in liver tissue (Figure 7 and Figure 9A). H1.4 is an isoform of histone H1 containing the consistent globular domain with nucleosome- and linker DNA-binding ability and an isoform-specific disordered N-/C-terminus [71]. Previous work indicates that H1.4 is generally present in most tissues and functionally redundant as no specific phenotype was identified in H1.4 knock-out mice [72,73,74,75]. Linker histone H1 (H1 variants) binds to the entry/exit sites of nucleosomes, which could be competitively occupied by MeCP2 in vitro [76,77,78], as was further confirmed by live cell fluorescence photobleaching and recovery assay [78]. Moreover, MeCP2 was more accumulated (upregulated) in brain PCH (Figure 7 and Figure 9A). Thus, we hypothesized a possible competitive relationship between H1 and MeCP2 in the PCH region. The MeCP2 level was higher in the mouse brain as compared to histone H1, which was generally expressed in both the brain and liver (Figure 9B). Yet, both H1 and MeCP2 locally accumulated at the PCH compartments in brain and liver slices (Figure 9C), although MeCP2 showed a much higher enrichment in brain PCH (Figure 9D and Figure S7).

Further, we examined the possible interplay between histone H1.4 and MeCP2 using cultured C2C12 cells with differential MeCP2 and H1.4 levels after transfection (Figure S10). Histone H1.4 and MeCP2 accumulated at PCH compartments in a level-dependent manner. Histone H1 enrichment at PCH was counteracted by increasing levels of MeCP2 and the same was true for MeCP2 enrichment at PCH in the presence of increasing amounts of histone H1.4 (Figure 9E). In addition, H1.4 and MeCP2 also exhibited opposite functions in PCH dynamics. Whereas H1.4 showed a minor ability to decrease the PCH compartment size, MeCP2, in contrast, promoted the PCH size enlargement (Figure 9F). Thus, we conclude that MeCP2 competes with histone H1 for PCH localization and compartmentalization.

## 4. Conclusions

Constitutive heterochromatin is involved in the maintenance of genome stability [37,79] and is required for nuclear spatial organization [12]. Dysfunction of heterochromatin has been associated with cancer progression [37]. Recently, constitutive heterochromatin was reported to constitute membraneless liquid-like compartments that concentrate some factors inside while excluding others, thus regulating nuclear DNA metabolism [80,81,82,83]. In mouse cells, constitutive heterochromatin can be found at pericentromeric regions, which cluster during interphase, forming pericentromeric heterochromatin (PCH) compartments [10,11,84]. PCH organization differs between cell types [39] and changes upon differentiation [15,17]. Thus, here, we addressed whether the observed changes in PCH organization are related to its proteomic composition.

The mouse brain and liver were chosen for the isolation of nuclei and PCH, followed by proteomic analysis, as mouse brain tissue showed significantly higher heterochromatin clustering (lower PCH numbers and larger volumes) in comparison to liver tissue (Figure 1). To purify the PCH compartments, we adapted a protocol for PCH isolation from mouse liver published by Prusov and Zatsepina [42,43]. Importantly, in contrast to other immunoprecipitation-based heterochromatin enrichment methods [60,61,62,63], this approach relies on the chromatin compaction levels and thus is not biased towards a selected and previously identified heterochromatin component. In addition, this protocol yields native heterochromatin as it does not require protein cross-linking.

For the mass spectrometry analysis, we used a dimethyl labeling approach, which allowed quantitative comparison of different tissue samples. To base our analysis only on the most reliable protein hits, we considered only proteins that were (1) reproducibly identified in all three biological replicates of both nuclei and PCH fraction (PCH-located proteins) and (2) highly abundant in PCH compared to nuclei (the ratio of intensities in PCH/nuclei > 1). However, we cannot rule out that we lost a fraction of PCH-enriched proteins that were not mainly localized (<50% of the protein molecules present at PCH) or weakly bound to the PCH. The hits with high abundance in both brain and liver PCH were recognized as PCH-enriched proteins, including several histones, DNA/chromatin proteins, and transcription regulators. However, nuclear membrane- and ribosome-related proteins were co-identified. As PCH localizes closely to nucleoli and the nuclear membrane [40,85,86], it is possible that the nuclear membrane and nucleoli were copurified and, thus, contaminated the isolated PCH fraction. Previous heterochromatin proteomic studies have also identified multiple RNA- and nuclear membrane-related proteins [60,87,88]. Thus, nuclear membrane and nucleolus contamination is likely to be a common issue during heterochromatin isolation and additional strategies should be adopted to compensate for it, regardless of the fact that nuclear membrane factors could also influence the PCH organization. Further, proteins that were highly abundant in brain PCH and in liver PCH, as well as proteins that were conserved in both tissues were identified based on the hit abundance in brain and liver PCH.

To validate the reliability of the data analysis pipeline, we examined the subcellular localization of several representative protein hits with nuclear membrane- or RNA-related functions and found that they did not accumulate at PCH compartments (Figure S5). Moreover, general chromatin proteins that distribute evenly across the nucleus were also picked up in our quantitative mass spectrometry analysis (Figure S8). However, a comparative analysis of mass spectrometry data showed that these PCH-unenriched proteins had a much lower protein abundance in PCH than those with PCH enrichment (Figure S9). Thus, the ATRX protein that was located at the boundary of actual PCH enrichment/non-enrichment was taken as the threshold for a further cut-off strategy, which we have called a validation-based cut-off (Figure 4D). Finally, 58 candidates were identified and considered as functional PCH-enriched proteins (Figure 7). The other candidates that were excluded after validation-based cut-off are probably either general chromatin-interacting proteins or located near the PCH. The reliability of this strategy was examined and confirmed by GO analysis (Figure 8). With GO analysis, terms like chromatin assembly and nucleosome positioning were very common, while terms associated with nucleoporins and nucleoli were absent. The localization of proteins classified into different categories (highly brain- or liver-abundant (upregulated) and PCH-conserved proteins) (Figure 7) was confirmed by immunofluorescence staining on mouse brain and liver tissue sections.

Topoisomerases and multiple histones and their variants (macroH2A.1, H2AX, and H3.3) were classified as conserved PCH candidates (Figure 7). On the one hand, topoisomerases are involved in opening chromatin to make it accessible for processes like transcription [89,90]; on the other hand, they have been described to be required for gene silencing of heterochromatic regions and chromosome condensation [91,92]. Further studies will be necessary to elucidate the role of the topoisomerases in pericentromeric heterochromatin in mammals, as most studies were performed in *Drosophila* and *Arabidopsis*, as the functions might be species-, cell type-, and differentiation-specific.

Compared to the canonical histone H2A, the histone variant macroH2A contains an additional large macro domain at its C-terminus, and the histone variant H2A.X contains a functional C-terminal Ser-Gln-Glu-Tyr motif [93]. MacroH2A.1 is involved in gene silencing and higher-order chromatin compaction, and it is associated with heterochromatin repeats [93,94,95], in line with our results. Although H2AX was described to distribute randomly on chromatin [96], another study in cancer cells found H2AX overrepresented at heterochromatin compared to euchromatin [97]. In addition, H2AX might be involved in maintaining genome stability [98], in chromatin remodeling, and in inactivation of sex chromosomes during meiosis in male mice [99]. Thus, we propose that H2AX accumulates at PCH in mouse brain and liver tissue, possibly playing a role in chromatin organization.

Histone H3.3 differs from the canonical H3 proteins by a few amino acid substitutions, one localized in the N-terminal tail, and the others in the histone-fold domain [93]. The variant replaces the canonical H3 at active genes and promoters but was also found at repetitive heterochromatin regions such as telomeres and pericentromeric heterochromatin [100]. In addition, it was reported to build a complex with ATRX, which is involved in H3.3 deposition at pericentromeric heterochromatin [100,101,102], which is in agreement with our results.

Linker histone H1.4 was identified as highly abundant (upregulated) in liver PCH (Figure 7). H1.4 is one of the main types of histone H1 [75]. The deletion of either H1.2, H1.3, or H1.4 suggested that the different variants can compensate for the lack of each other [74], but the differing distribution of the variants points toward variant-specific functions [75,103]. Sequential inactivation of all three variants resulted in a lower H1 to nucleosome ratio, reduced DNA packaging, and changes in gene expression [104,105].

MeCP2 and ATRX were identified as highly abundant (upregulated) in brain PCH (Figure 7). MeCP2 binds to methylcytosine on the DNA and modulates transcriptional regulation [106,107,108,109] and chromatin organization (reviewed in [110]). MeCP2 levels differ between cell types [111,112] and increase upon differentiation [15,111,112,113], which results in higher heterochromatin clustering [15,17,114]. More recently, we and others found that MeCP2 can modulate the PCH compartment dynamics via liquid–liquid phase separation, highlighting the functional importance of MeCP2 in PCH compartmentalization via multiple functions. Interestingly, coimmunoprecipitation experiments indicated an interaction of MeCP2 with ATRX [67,68], both of which were highly expressed in brain tissue. ATRX was reported to bind to tandem repeat sequences and to be involved in chromatin organization and subsequent gene silencing [115,116,117]. Moreover, ATRX might contribute to MeCP2-mediated heterochromatin organization during neural differentiation [118].

As MeCP2 and histone H1 were classified into distinct abundance groups in the brain and liver (Figure 7 and Figure 9A), we wondered if the two regulate PCH dynamics differentially. From previous studies, histone H1 and MeCP2 were reported to compete for binding to an overlapping chromatin binding site, as MeCP2 accelerated H1 exchange and both showed similar nucleosome binding motifs in vitro [78]. In vivo, the estimated amount of H1 was 0.45 and 1 molecules/nucleosome in neurons and glial cells, respectively [77]. In contrast, the amount of MeCP2 was 0.5 and 0.06 molecules/nucleosome in neurons and glial cells, respectively [76]. In addition, Skene et al. found a 2-fold increase in histone H1 levels in MeCP2-deficient neuronal nuclei, suggesting that MeCP2 can replace histone H1 in neurons [76]. Here, we found that MeCP2 and H1 repress the PCH enrichment of each other and exhibit opposite functions in PCH organization in cultured C2C12 cells. Thus, the distinct PCH organization we found in the brain versus the liver might be due to their differential protein abundance.

Of note, the heterochromatin protein 1 (HP1), which was used for heterochromatin enrichment in previous studies (e.g., [87]), was not reproducibly found to be enriched in the PCH fractions in our study. Thus, the HP1 was filtered out during the early stage of data analysis (Figure 4C). In line with our results, HP1 isoforms were reported to show a different localization dependent on the species, cell type, cell cycle, differentiation status, and isoforms (HP1α, HP1β, and HP1γ) [114,119,120,121]. HP1 also exhibits weak interactions with heterochromatin, as in fluorescence photobleaching assays, the HP1 fluorescence recovered much faster than MeCP2 [19,78]. As such, the HP1 was probably washed out during the PCH isolation steps as no cross-linking step was included.

In conclusion, the unbiased heterochromatin isolation procedure followed by subsequent quantitative mass spectrometry analysis and in vivo validation-based cut-off strategy described here was successfully used to elucidate the mouse pericentromeric heterochromatin proteome. Linker histone H1 and MeCP2 were found to be differentially enriched in liver and brain tissue, respectively. Subsequent functional analysis indicated that their competitive chromatin binding together with their opposing effect on the higher-order organization of chromatin are involved in the differing heterochromatin architecture in mouse brain and liver tissues. Further work should focus on (i) elucidating the proteome composition of other tissues; (ii) the functional analysis of candidates identified and their role in heterochromatin architecture; and (iii) the interplay between heterochromatin composition, architecture, and function.

## Data Availability

The raw proteomic data have been deposited at PRIDE (accession number: PXD045223).

## Supplementary Materials

The following supporting information can be downloaded at: https://www.mdpi.com/article/10.3390/cells13020139/s1, Figure S1: Centromeric heterochromatin organization in mouse tissues; Figure S2: Electron microscopy characterization of pericentromeric heterochromatin organization in mouse tissues; Figure S3: PCH enriched proteins in mouse tissues identified by quantitative mass spectrometry; Figure S4: Gene ontology (GO) analysis of the PCH enriched proteins; Figure S5: Localization of PCH enriched proteins with GO terms for nuclear membrane, splicing or transcription; Figure S6: Nuclear abundance of PCH enriched proteins with GO terms for nuclear membrane, splicing or transcription; Figure S7: Workflow for nuclei and PCH segmentation after immunofluorescence staining; Figure S8: Candidates with GO terms for chromatin but no PCH accumulation in mouse brain and liver; Figure S9: Cut-off strategy based on immunofluorescence staining and localization of candidates; Figure S10: MeCP2 competes with histone H1.4 at PCH; Table S1: Oligonucleotide characteristics; Table S2: Primary and secondary antibody characteristics; Table S3: Eukaryotic cell line characteristics; Table S4: Instrument and imaging system characteristics; Table S5: Plasmid characteristics; Table S6: Plot statistics Figure 3E, Figure 5B and Figure 9D; Table S7: Heatmap plot statistics Figure 9E,F; Table S8: Plot statistics Figure S2; Table S9: Proteins enriched in heterochromatin identified by mass spectrometry.
