# Supplementary material for: The Proteomic Composition and Organization of Constitutive Heterochromatin in Mouse Tissues
Source: Cells. 2024 Jan 11;13(2):139. doi: 10.3390/cells13020139 (PMC10814525; doi:10.3390/cells13020139)
Supplement: Supplementary file 1 [file cells-13-00139-s001.zip › cells-2723439-supplementary.pdf]

Supplementary information

Supplementary Figures

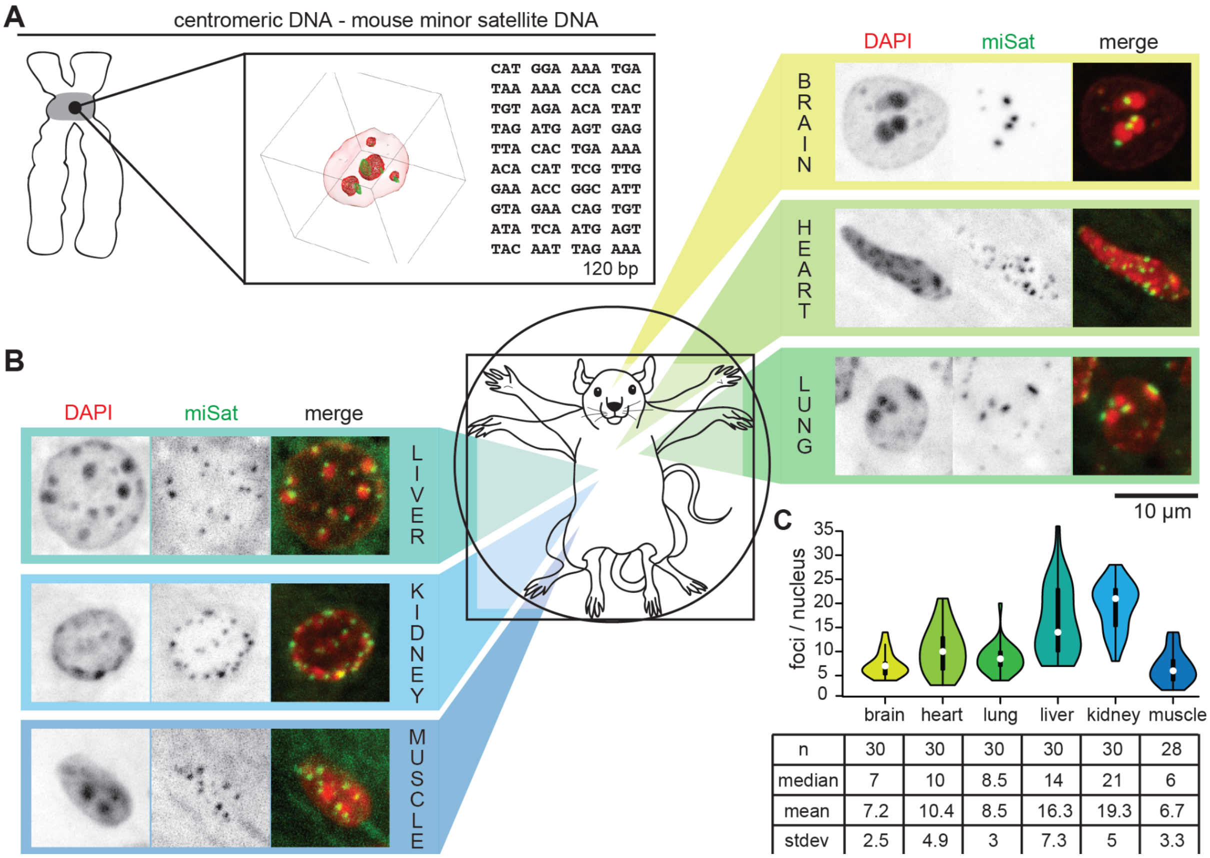

	brain	heart	lung	liver	kidney	muscle
n	30	30	30	30	30	28
median	7	10	8.5	14	21	6
mean	7.2	10.4	8.5	16.3	19.3	6.7
stdev	2.5	4.9	3	7.3	5	3.3

Figure S1. Centromeric heterochromatin organization in mouse tissues.

(A) Rotated view of 3D projection of minor satellite (centromeric) DNA fluorescence in situ hybridization (FISH; green) overlaid with the DNA stained with DAPI (red) and minor satellite DNA repeat sequences. (B) Minor satellite (miSat) DNA FISH (green) overlaid with DNA (red) in different mouse tissues as indicated. (C) Quantitation of minor satellite foci in different mouse organs. Violin plots represent the median foci number (white mark), spread (line; upper and lower quartile), the whiskers the 95 % interval and the rotated kernel density plot all possible values. Corresponding statistics for minor satellite foci number per nucleus was calculated by Volocity software after segmenting single foci.

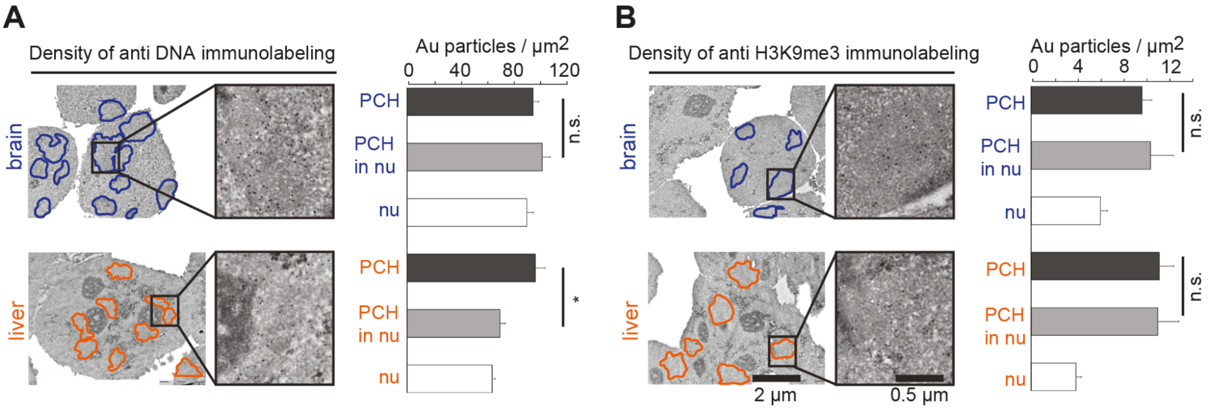

Figure S2. Electron microscopy characterization of pericentromeric heterochromatin organization in mouse tissues.

Immunolabeling using DNA (A) and H3K9me3 (B) antibodies and 10 nm colloidal gold particles-coupled secondary antibodies on ultrathin sections of isolated nuclei prepared from mouse liver and brain cells. Blue or orange lines encircle the area of the PCH. The right sides of the images represent the enlargement of a chosen PCH (marked by a black frame) showing the distribution of antibodies in the PCH region. Graphs showing colloidal gold immunolabeling density (Au particles/µm2) for DNA and H3K9me3 antibodies in each type of sample: nuclei (nu), PCH in isolated nuclei (PCH in nu) and isolated PCH (PCH) from both liver and brain cells. Gold nanoparticles were detected and counted using the ImageJ plug-in ThunderSTORM. Error bars: Mean ± SEM, N = 11 – 17. The statistics are summarized in table S8.

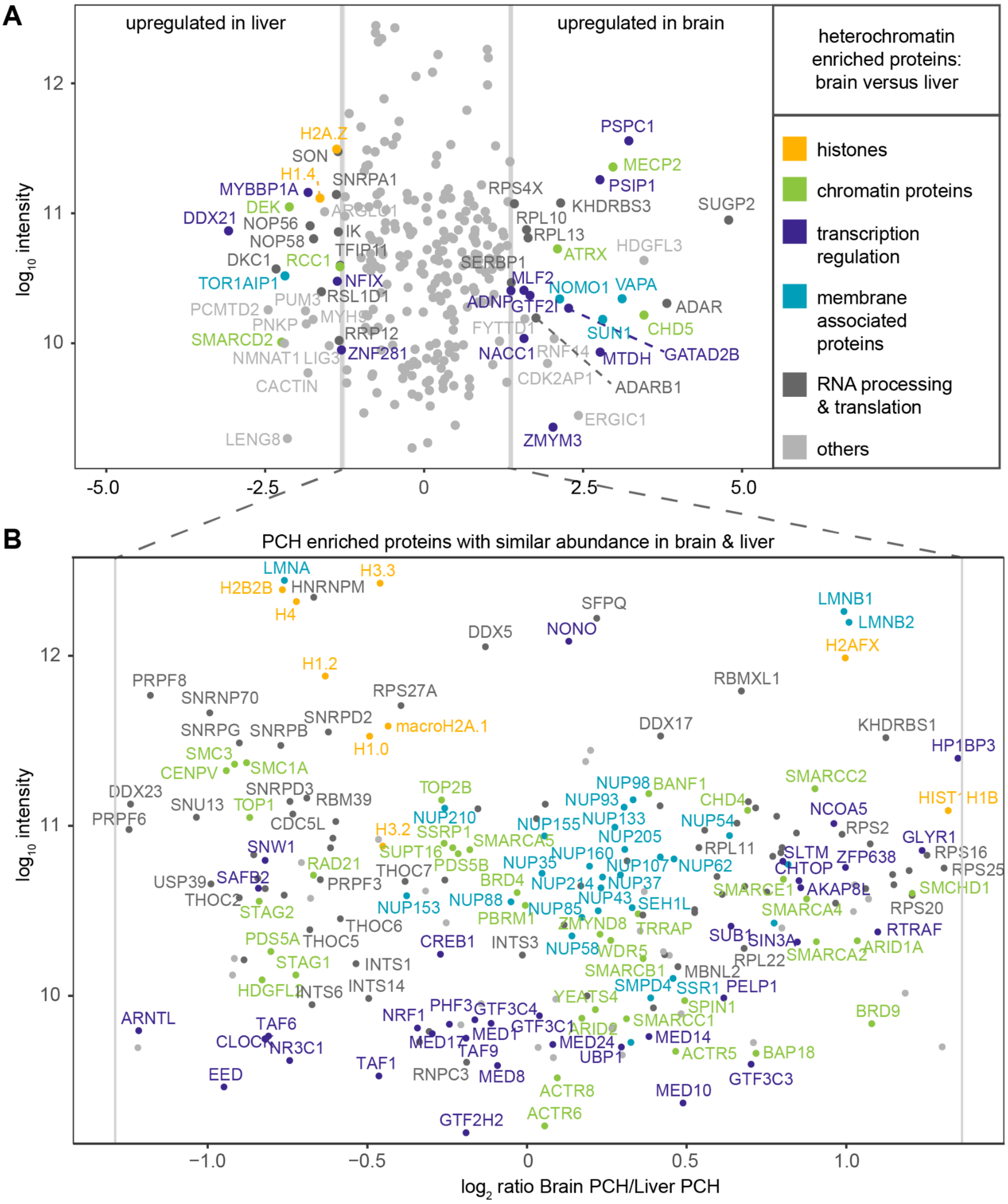

Figure S3. PCH enriched proteins in mouse tissues identified by quantitative mass spectrometry.

All proteins identified in three biological replicates and enriched in the PCH compared to the nuclei (see table S9) were used as input (Figure 4C). (A) The dot plot shows the log10 intensity plotted against the log2 ratio of brain PCH versus liver PCH. Proteins with equal abundance are depicted in gray, and proteins highly enriched in the brain or liver PCH (upper or lower 10%) were labeled and color-coded. The color code indicates the protein function manually assigned based on the UniProt webpage functional information. Histones are labeled in orange, chromatin proteins in green, proteins involved in transcriptional regulation in dark blue, membrane associated proteins in cyan, proteins involved in RNA processing or RNA-binding proteins in dark gray, and proteins not fitting into the categories in light gray. (B) Rescaled version of the dotplot shown in (A) focusing on the heterochromatin enriched proteins with similar abundance between brain and liver PCH. Color coding is as described in (A).

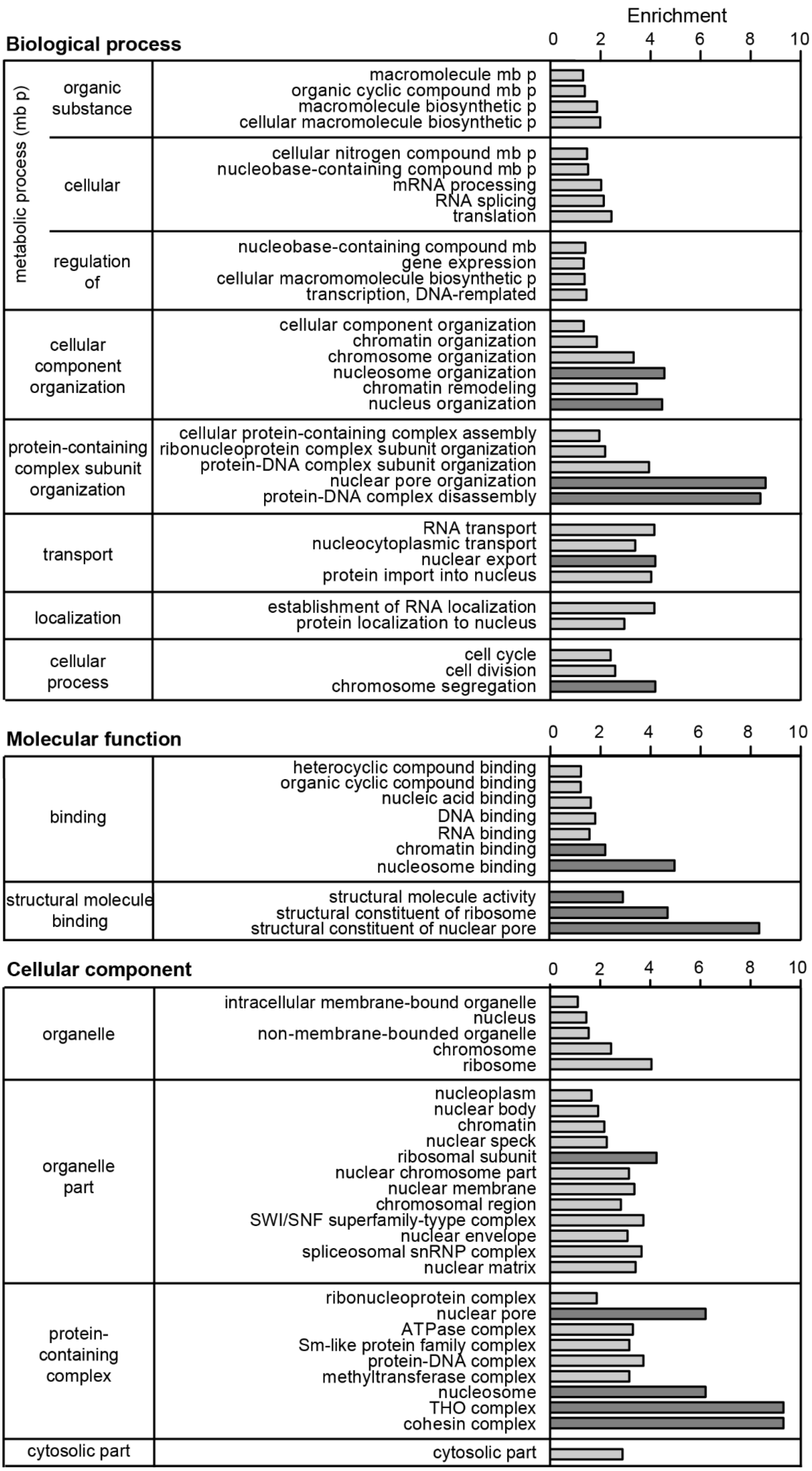
Enrichment
Biological process
0 2 4 6 8 10
metabolic process (mb p)
organic substance
macromolecule mb p
organic cyclic compound mb p
macromolecule biosynthetic p
cellular macromolecule biosynthetic p
cellular
cellular nitrogen compound mb p
nucleobase-containing compound mb p
mRNA processing
RNA splicing
translation
regulation of
nucleobase-containing compound mb
gene expression
cellular macromomolecule biosynthetic p
transcription, DNA-remplated
cellular component organization
cellular component organization
chromatin organization
chromosome organization
nucleosome organization
chromatin remodeling
nucleus organization
protein-containing complex subunit organization
cellular protein-containing complex assembly
ribonucleoprotein complex subunit organization
protein-DNA complex subunit organization
nuclear pore organization
protein-DNA complex disassembly
transport
RNA transport
nucleocytoplasmic transport
nuclear export
protein import into nucleus
localization
establishment of RNA localization
protein localization to nucleus
cellular process
cell cycle
cell division
chromosome segregation
Molecular function
0 2 4 6 8 10
binding
heterocyclic compound binding
organic cyclic compound binding
nucleic acid binding
DNA binding
RNA binding
chromatin binding
nucleosome binding
structural molecule binding
structural molecule activity
structural constituent of ribosome
structural constituent of nuclear pore
Cellular component
0 2 4 6 8 10
organelle
intracellular membrane-bound organelle
nucleus
non-membrane-bounded organelle
chromosome
ribosome
organelle part
nucleoplasm
nuclear body
chromatin
nuclear speck
ribosomal subunit
nuclear chromosome part
nuclear membrane
chromosomal region
SWI/SNF superfamily-tyype complex
nuclear envelope
spliceosomal snRNP complex
nuclear matrix
protein-containing complex
ribonucleoprotein complex
nuclear pore
ATPase complex
Sm-like protein family complex
protein-DNA complex
methyltransferase complex
nucleosome
THO complex
cohesin complex
cytosolic part
cytosolic part

Figure S4. Gene ontology (GO) analysis of the PCH enriched proteins.

All PCH enriched protein hits in both brain and liver were used as input (Figure S3). The protein list was subjected to the GOrilla tool [53] for gene ontology analysis in the categories biological process, molecular function, and cellular component. The proteins enriched in heterochromatin were added as the target list, all identified proteins (without cut-off or filtering) as the background list. GO terms with a p-value lower than 5 x 10-5 were considered. The GO terms were grouped according to a common gene ontology term within the diagram of the GOrilla output and redundant terms were removed manually. Plotted is the number of genes in the GO term (b) as % of total genes in the target list (n) and the enrichment calculated by the GOrilla tool as (b/n)/(B/N) with b: number of genes in the target list associated with specific GO term; n: total number of genes in the target list; B: number of genes in the background list associated with specific GO term; N: total number of genes in the background list. The proteins with the highest enrichment in each category were highlighted in dark gray.

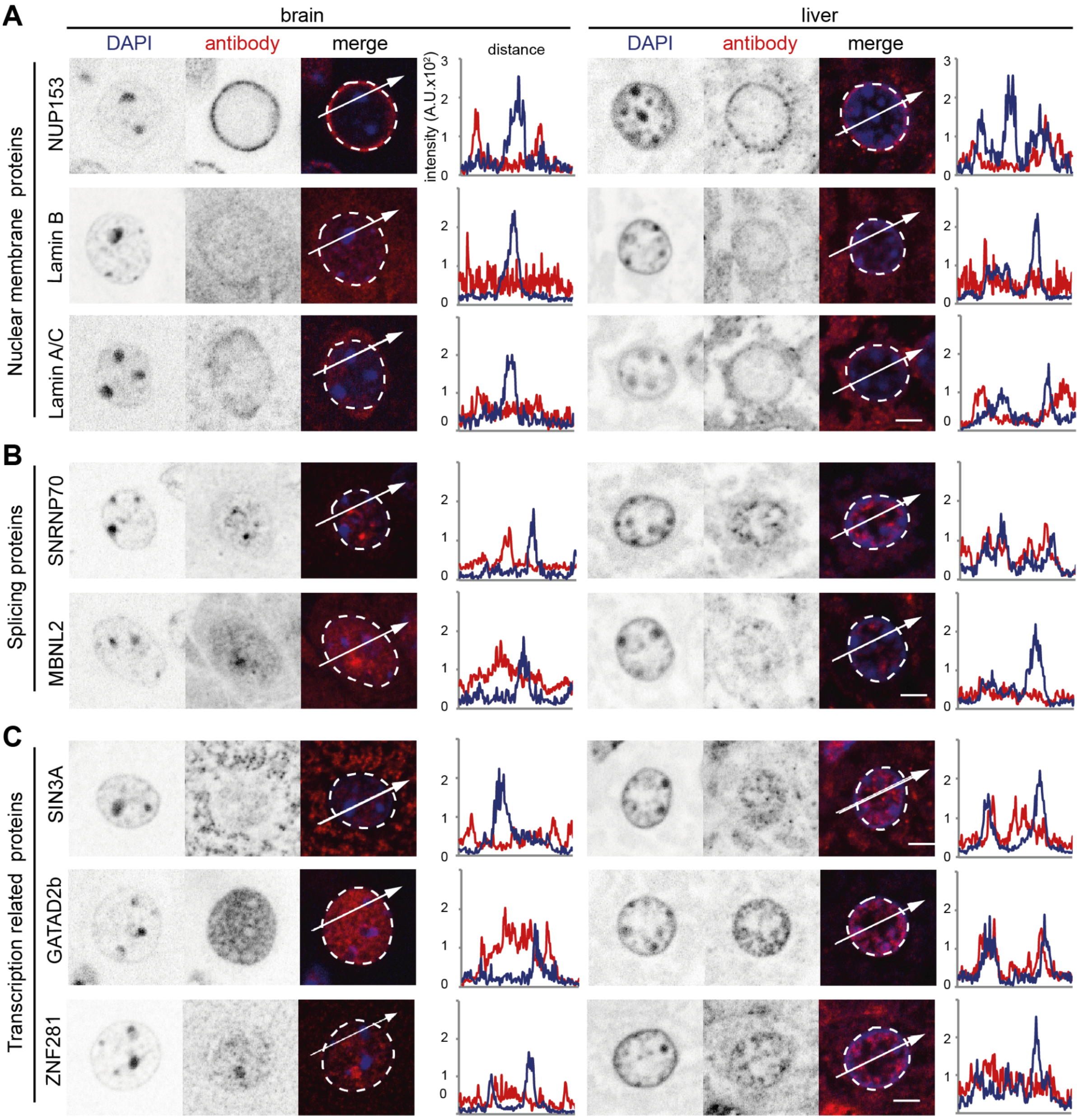

Figure S5: Localization of PCH enriched proteins with GO terms for nuclear membrane, splicing or transcription.

The candidates enriched to GO terms of nuclear membrane (A), splicing (B), or transcription regulation (C) were detected on mouse brain and liver tissue slices by immunofluorescence staining. The nuclear outlines are marked in white on the merged channel image. Line plots of the fluorescence intensity in arbitrary units (A.U.) plotted against the distance depict the colocalization of the antibody staining (red) with the DNA counterstain (DAPI, blue). Scale bar 5 µm.

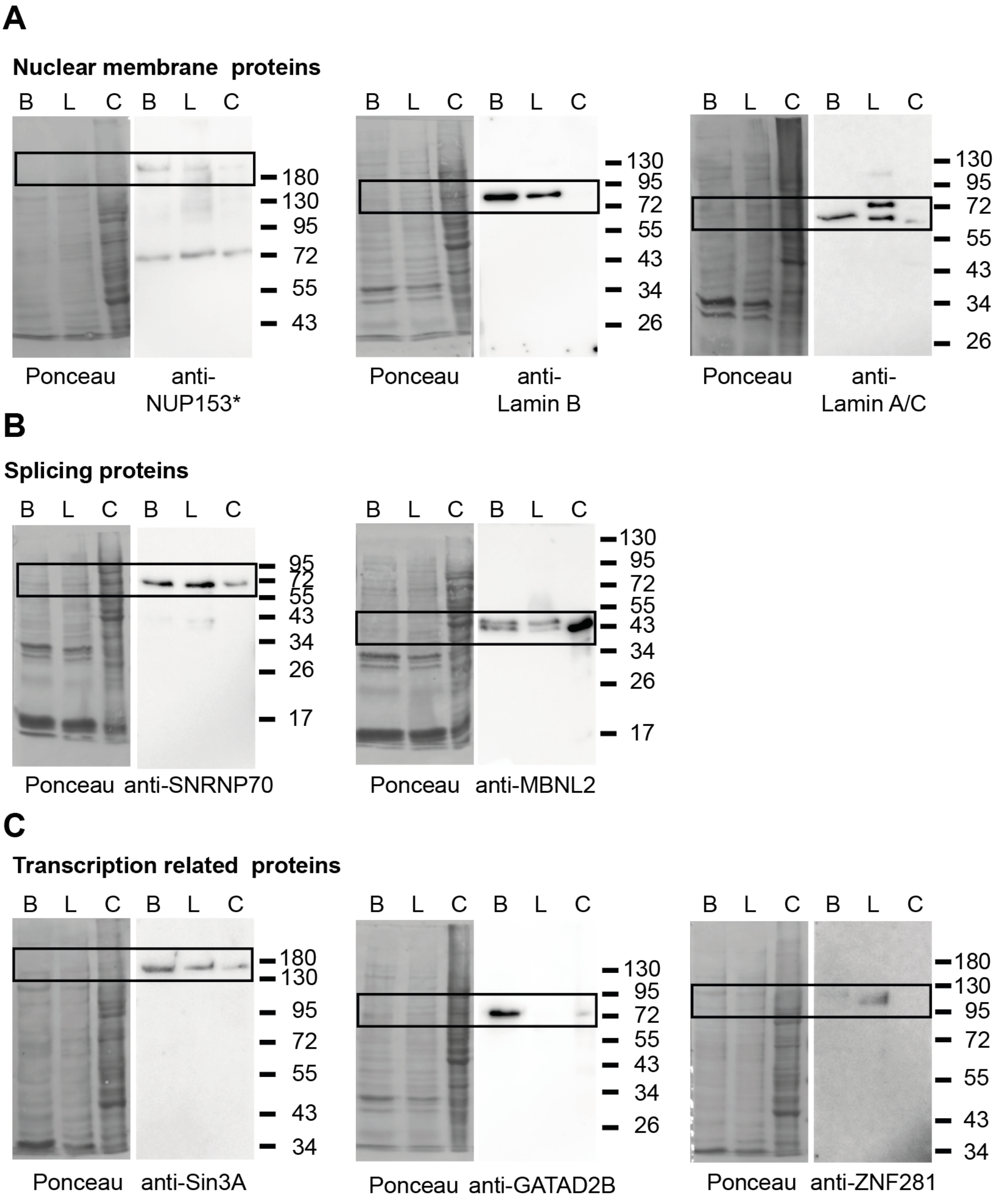

Figure S6. Nuclear abundance of PCH enriched proteins with GO terms for nuclear membrane, splicing or transcription.

The candidates enriched to GO terms of nuclear membrane (A), splicing (B), or transcription regulation (C) as shown in Figure S5 were detected on mouse brain and liver tissues by western blot analysis. B: brain nuclei lysate, L: liver nuclei lysate, C: whole cell lysate from mouse myoblasts. The protein transfer efficiencies were validated by Ponceau S staining on the left, and the antibody signals with chemiluminescence detection were shown on the right. The molecular weight marker indicates the protein weight in kDa and the black boxes mark the bands of interest.

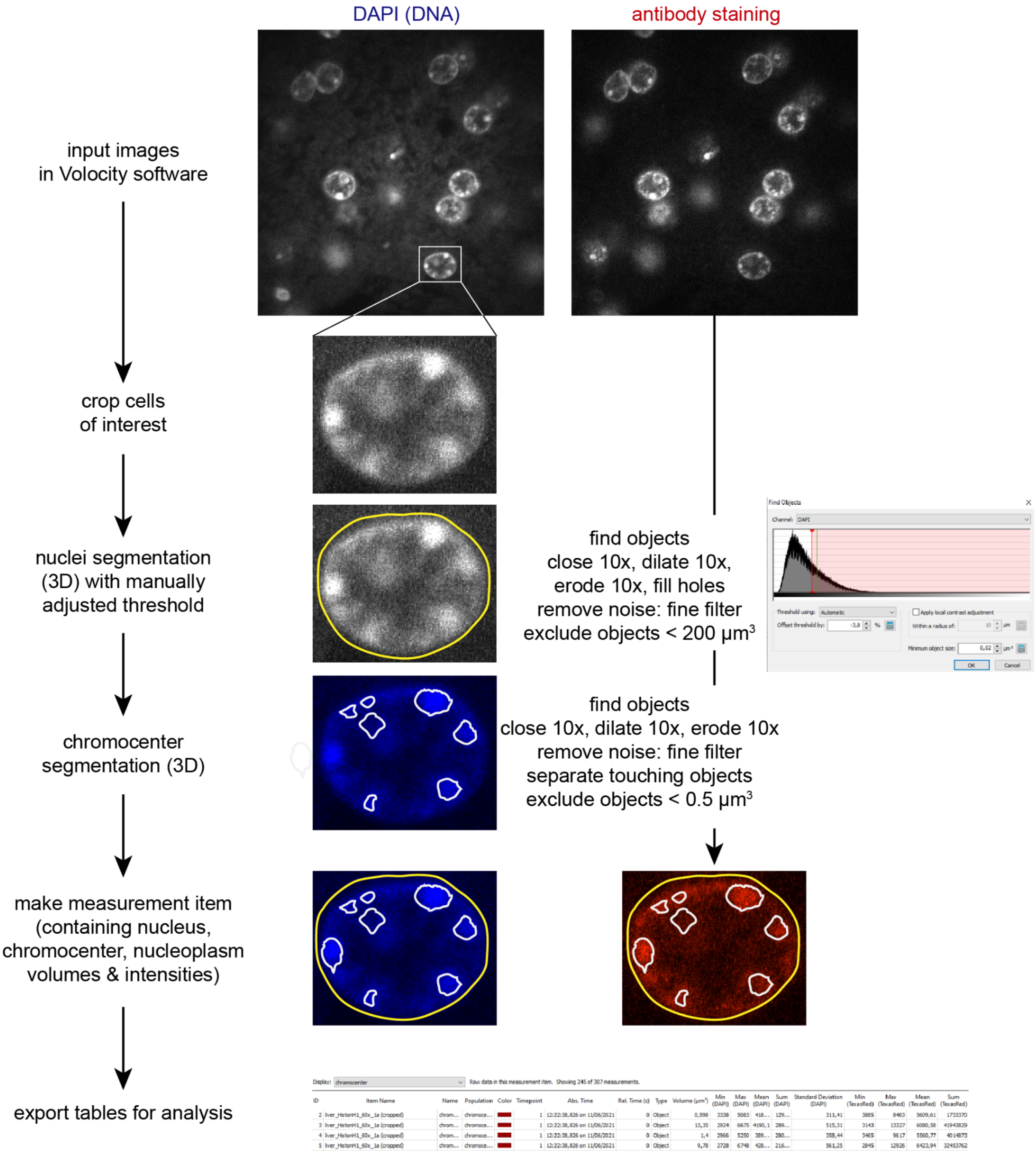

Figure S7. Workflow for nuclei and PCH segmentation after immunofluorescence staining.

The confocal z-stacks were obtained on an Ultra-Vox spinning disk system automatically saving the images in the Volocity software (Perkin Elmer). Based on the DAPI channel, individual cells were cropped, nuclei and PCH were segmented with the parameters described and a measurement item was created for each cell. The results were exported as tables for further analysis using the R software package.

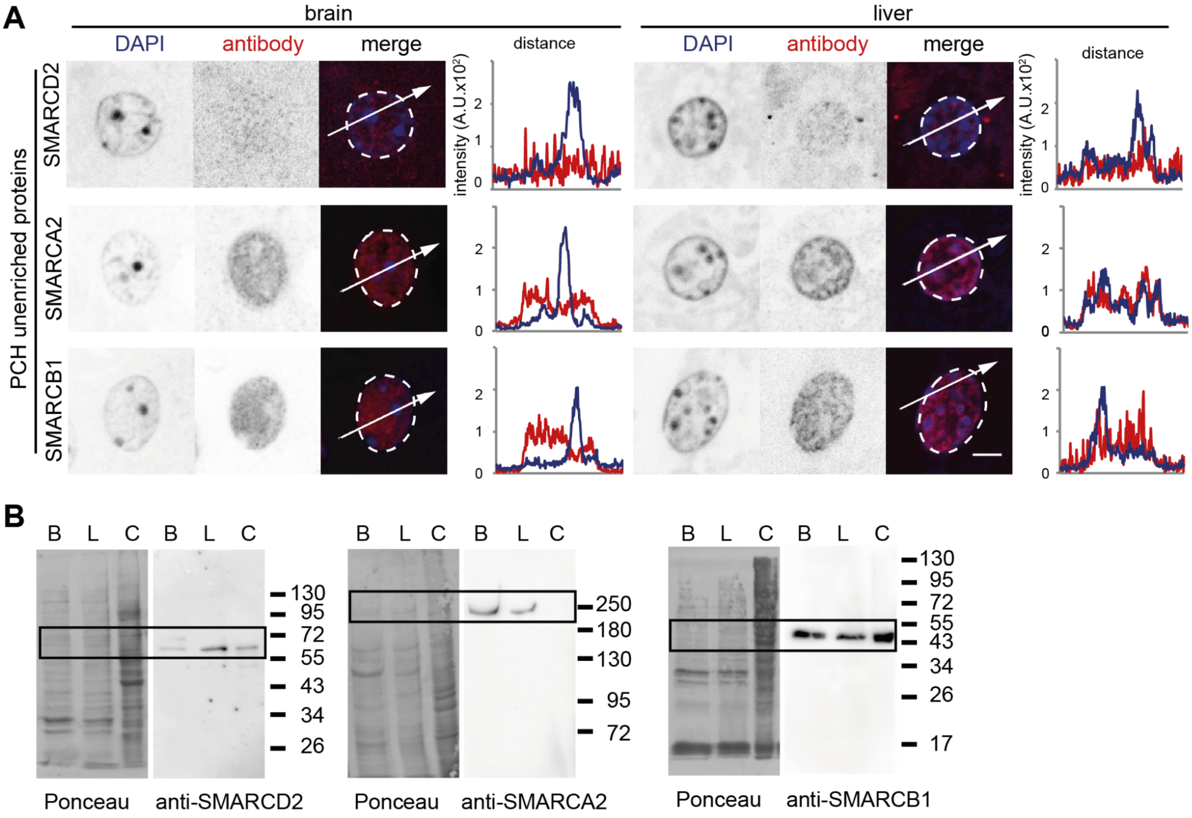

Figure S8. Candidates with GO terms for chromatin but no PCH accumulation in mouse brain and liver.

The candidates enriched for GO terms for chromatin were detected on mouse brain and liver tissue slices and the candidates with no PCH accumulation in both mouse brain and liver tissues were shown. (A) Immunofluorescence staining on mouse brain and liver tissue slices. The nuclear outlines are marked in white on the merged channel image. Line plots of the fluorescence intensity in arbitrary units (A.U.) plotted against the distance depict the colocalization of MeCP2 or H1 (red) with the DNA counterstain (DAPI, blue). Scale bar 5 µm. (B) Western blot analysis to detect the protein levels of proteins examined in (A) in brain and liver nuclei. B: Brain nuclei lysate; L: liver nuclei lysate; C: whole mouse myoblast cell lysate.

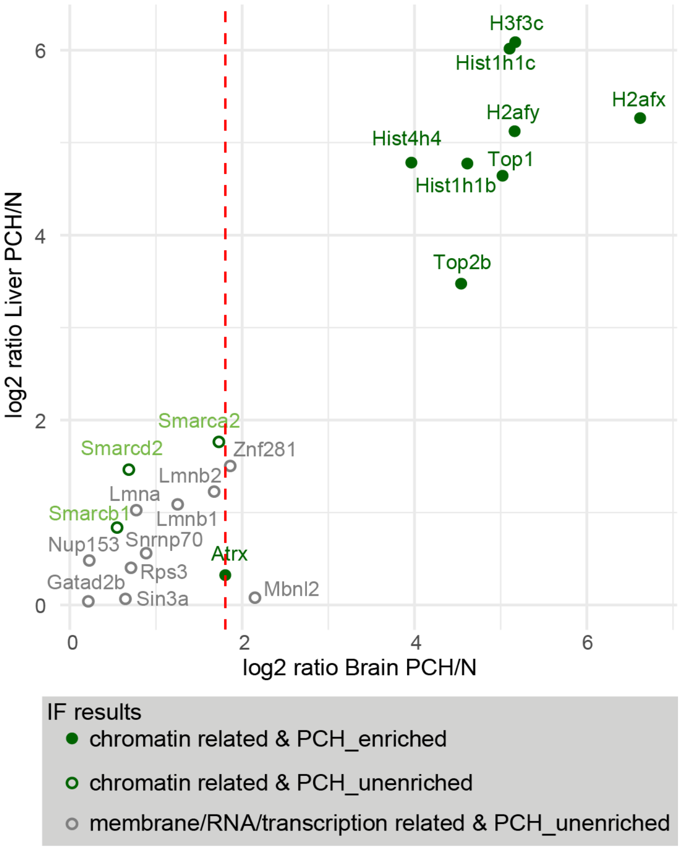

Figure S9. Cut-off strategy based on immunofluorescence staining and localization of candidates.

The candidates examined above (18 proteins) (Figure 5-6, Figure S5-S6, and Figure S8) were extracted from all PCH accumulated candidates (Figure S3). The dot plot shows the log2 ratio of protein intensities in liver PCH versus liver nuclei (N) plotted against the log2 ratio of protein intensities in brain PCH versus brain nuclei (N). The 18 candidates were classified into chromatin related (green circle) and chromatin unrelated (membrane, RNA, or transcription related) (gray circle) according to their GO terms (Figures S3-S4). The candidates were classified into PCH enriched (solid circles) and unenriched (hollow circles) based on the immunofluorescence staining (Figure 5, Figure S5 and Figure S8). Dashed red line represents the log2 ratio of ATRX intensities in brain PCH versus brain nuclei.

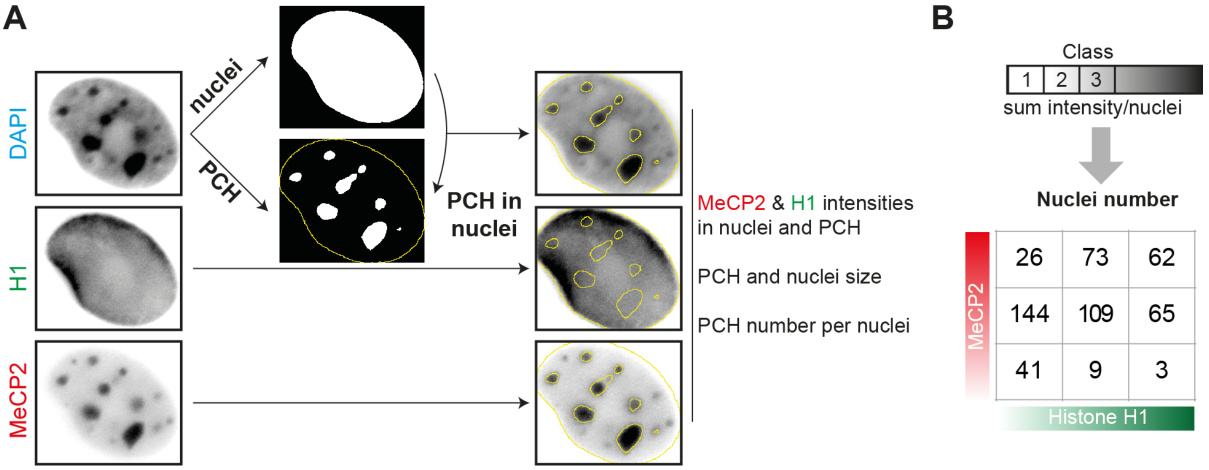

Figure S10: MeCP2 competes with histone H1.4 at PCH

(A) Workflow to analyze the MeCP2 and H1.4 levels in PCH of cultured C2C12 cells. The confocal images were obtained on the Nikon Crest spinning disk system and analyzed using FIJI software. The cell nuclei and corresponding PCH were recognized based on the DAPI intensities. The nuclei and PCH size, MeCP2 and H1.4 intensities in both nuclei and PCH, and PCH compartment numbers per nuclei were measured. (B) The cells were subgrouped into four groups based on the sum intensities after removing cells with extremely high or low signals. The cell number in each group was listed.

Supplementary tables

Table S1: Oligonucleotide characteristics

Name	Sequence [5' → 3']	Use	Reference
MajS-F	AAAATGAGAAACATCCACTTG	PCR	Frauer et al., 2011 [54]
MajS-R	CCATGATTTTCAGTTTTCTT	PCR	Frauer et al., 2011 [54]
MinS-F	CATGGAAAATGATAAAAACC	PCR	Lehnertz et al., 2003 [55]
MinS-R	CATCTAATATGTTCTACAGTGTG	PCR	Lehnertz et al., 2003 [55]

Table S2: Primary and secondary antibody characteristics

Reactivity	Host	Dilution	Application	Cat #	Company / Reference
Anti-ATRX (D-5)	mouse	1:50	IF / WB	sc-55584	Santa Cruz Biotechnology
Anti-mC	mouse	1:100	slot blot	MMS-900P-B	Eurogentec
Anti-DNA	mouse	1:50	EM	CBL 61014	Progen
anti-B23	mouse	1:500	WB	B0556	Sigma
Anti-Gatad2b	rabbit	1:200	IF / WB	AB-2641884	invitrogen
Anti-Histone H1	rabbit	5 µg/ml0	IF / WB	sc-8030	Santa Cruz
Anti-Histone macro H2A.1	rabbit	1:200 / 1:500	IF / WB	07-219	upstate
Anti-Histone H2AX	rabbit	1:200	IF	A300-083A	Bethyl
Anti-Histone H2AX	rabbit	1:1000	WB	ab20669	abcam
Anti-Histone H3.3 (EPR17899)	rabbit	1:1000	IF / WB	ab176840	abcam
anti-H3K9me3	rabbit	1:350	WB	39161	Active Motif
Anti-Histone H4	rabbit	1:200	IF / WB	ab7311	abcam
Anti-Lamin A/C (XB 10)	mouse	Undiluted TCSN	IF / WB	-	gift from Brian Burke
Anti-Lamin B (X223)	mouse	Undiluted TCSN	IF / WB	65147C	Progen
Anti-Mbnl2 (3B4)	mouse	1:50	IF / WB	sc-136167	Santa Cruz Biotechnology
Anti-MeCP2 (4H7)	rat	undiluted TCSN	IF / WB	-	Jost et al., 2011
Anti-mSin3A (K20)	rabbit	1:50	IF / WB	sc-994	Santa Cruz Biotechnology
Anti-Nup153 (QE5)	mouse	1:100	IF / WB	ab24700	abcam
Anti-SMARCA2/BRM	rabbit	1:100 / 1:1000	IF / WB	ab15597	abcam
Anti-SMARCB1/BAF47 (D8M1)	rabbit	1:250 / 1:1000	IF / WB	91735S	Cell Signaling Technology
Anti-Smarcd2 (EPR20860-251)	rabbit	1:100 / 1:1000	IF / WB	ab220164	abcam
Anti-Topoisomerase I (EPR5375)	rabbit	1:100	IF / WB	ab109374	abcam
Anti-Topoisomerase II alpha + beta (TOP2B) (EPR5377)	rabbit	1:100 / 1:1000	IF / WB	ab109524	abcam
anti-tubulin	mouse	1:5000	WB	T9026	Sigma
Anti-U1 snRNP70 (C-3)	mouse	1:50 / 1:100	IF / WB	sc-390899	Santa Cruz Biotechnology
Anti-Znf281 (D-8)	mouse	1:50 / 1:100	IF / WB	sc-166933	Santa Cruz Biotechnology
Anti-Halo	mouse	1:1000	IF	G921A	Promega GmbH
Anti-mouse IgG Cy3	donkey	1:500	IF	715-166-151	Jackson ImmunoResearch Laboratories, inc.
anti-mouse IgG Cy5	donkey	1:250	IF	715-175-150	Jackson ImmunoResearch Laboratories, inc.
Anti-mouse IgG HRP	sheep	1:5000	WB	NA 931	Amersham Pharmacia Biotech

anti-rabbit IgG Cy3	donkey	1:500	IF	711-165-152	Jackson ImmunoResearch Laboratories, inc.
anti-rabbit IgG Cy5	donkey	1:400	IF	711-175-152	Jackson ImmunoResearch Laboratories, inc.
Anti-rabbit IgG HRP	goat	1:10000	WB	A0545	Sigma-Aldrich, Inc.
anti-mouse IgG colloidal gold coupled	goat	1:20	EM	810.022	Aurion
anti-rabbit IgG colloidal gold coupled	goat	1:20	EM	810.011	Aurion
Anti-rat IgG Cy5	donkey	1:250	IF	712-175-153	Jackson ImmunoResearch Laboratories, inc.

IF: immunofluorescence; WB: Western blot; HRP: horseradish peroxidase; TCSN: Tissue culture supernatant; EM: electron microscopy.

Table S3: Eukaryotic cell line characteristics

Name	Species	Type	Genotype	Reference
C2C12	Mus musculus	myoblast	wildtype	Yaffe et al., 1977 [128]

Table S4: Instrument and imaging system characteristics

Microscope/ Company	Lasers/lamps	Filters (ex. & em. (nm))	Objectives/ lenses	Detection system	Application
Widefield Axiovert 200 / Zeiss	HBO100 mercury lamp	DAPI (300-400 & 410-510)	40x Plan-Neofluar NA 1.4 Oil Ph3	12-bit AxioCam mRM	Fluorescence imaging PCH isolation steps
Widefield Axioplan / Zeiss	HBO100 mercury lamp	DAPI (450-490 & 515-565)	40x Plan-Neofluar NA 1.3 Oil Ph3	12-bit AxioCam mRM	Fluorescence imaging PCH isolation steps
UltraView VoX spinning disk on an inverted Nikon Ti-E microscope / PerkinElmer	Solid state diode lasers (405 nm, 561 nm, 640 nm)	405/568/640 405: 415–475 561: 580–650 640: 664–754	60x Plan-Apochromat NA 1.45 Oil	cooled 14-bit Hamamatsu® C9100-50 EMCCD	Confocal z-stack imaging of tissue slices
CREST X-light V2 spinning disk on an inverted Nikon Eclipse TiE2/PerkinElmer	SPECTRA X LED 470/24 nm (196 mW) 640/30 nm (231 mW)	em.: Quadbandpass (432/25 nm ; 515/25 nm; 595/25 nm; 730/70 nm)	CFI Apo TIRF DIC 63x NA 1.49 Oil	Nikon Qi2 751600 16.25 MPx	Confocal imaging of cultured cells
Amersham AI600 imager / GE Healthcare	Chemiluminescence	-	-	16-bit Peltier cooled Fujifilm Super CCD	Western Blot imaging
FEI Morgagni TEM	80 kV in FEI	-	-	CCD camera MegaView III	Electron microscopy

Table S5: Plasmid characteristics

Name	pc number	Fluorophore	Gene species	Promotor	Reference
phMeCP2-halo	pc3972	Halo	human	CMV	this study
pEGFP-Histone H1.4	pc2378	EGFP	human	CMV	Th'ng et al., 2005 [57]

Table S6: Plot statistics Figures 3E, 5B and 9D

Figure	Sample	n	Median	Mean	Stdev	95% CI	p-value
3E	Mean mC intensity in:						
	brain PCH	60	2841.5	2867.5	365.6	-	-
	brain euchromatin	60	2036.1	2049.8	111.3	-	-
	liver PCH	60	2899.8	2885.5	330.4	-	-
	liver euchromatin	60	2343.2	2389.6	296.4	-	-
5B	ATRX brain	30	1.2	1.22	0.11	-	1.11E-11
	ATRX liver	32	1.05	1.06	0.03	-	
	TopI brain	30	1.21	1.26	0.12	-	3.96E-7
	TopI liver	31	1.42	1.42	0.07	-	
	TopII brain	35	1.4	1.42	0.28	-	2.35E-07
	TopII liver	32	1.09	1.09	0.02	-	
	Histone H4 brain	33	1.21	1.21	0.1	-	8.71E-10
	Histone H4 liver	32	1.43	1.43	0.15	-	
	Histone 3.3 brain	31	1.24	1.26	0.14	-	0.8102
	Histone 3.3 liver	33	1.27	1.26	1.1	-	
	macro H2A.1 brain	35	1.33	1.32	0.12	-	8.10E-11
	macro H2A.1 liver	32	1.15	1.15	0.05	-	
	Histone H2AX brain	34	1.27	1.29	0.15	-	1.81E-07
	Histone H2AX liver	31	1.13	1.14	0.05	-	
9D	Histone H1 brain	30	1.52	1.66	0.47	-	
	Histone H1 liver	31	1.47	1.52	0.28	-	0.3934
	MeCP2 brain	30	1.68	1.69	0.26	-	
	MeCP2 liver	30	1.02	1.02	0.01	-	< 2.2e-16

Table S7: Heatmap plot statistics Figure 9E-F

H1 classes	MECP2 classes	PCH mean area	H1 in PCH	MeCP2 in PCH
			Mean intensity	Mean intensity
1	1	1.19	400.35	885.12
2	1	1.16	406.83	858.12
3	1	1.15	428.28	779.47
1	2	1.22	399.64	1101.32
2	2	1.21	403.89	1065.37
3	2	1.21	410.43	1000.15
1	3	1.34	402.92	1374.78
2	3	1.32	404.16	1317.89
3	3	1.27	407.48	1275.18

Table list the mean values for heatmap plotting

Table S8: Plot statistics Figures S2

Sample	n	Median	Mean	Stdev	95% CI	p-value
anti-DNA Au particles in liver:						
PCH isolated	13	-	97.2	26.2	-	
PCH in nuclei	11	-	70.4	13.8	-	< 0.05
nuclei	11	-	64.4	7.5	-	
anti-DNA Au particles in brain:						
PCH isolated	13	-	95.4	15.1	-	
PCH in nuclei	14	-	102.3	22.6	-	n.s.
nuclei	14	-	90.8	18.1	-	
anti-H3K9me3 Au particles in liver:						
PCH isolated	16	-	11.2	5.0	-	
PCH in nuclei	16	-	11.1	7.4	-	n.s.
nuclei	16	-	3.9	1.9	-	
anti-H3K9me3 Au particles in brain:						
PCH isolated	13	-	9.7	3.1	-	
PCH in nuclei	17	-	10.4	8.3	-	n.s.
nuclei	17	-	6.1	2.4	-	

Table S9: Proteins enriched in heterochromatin identified by mass spectrometry.

Protein symbol	Protein ID	log10 intensity	log2 ratio BC/LC	protein function	enrichment category
ACTA2	P62737	10.57	1.07	others	common
ACTR5	Q80US4	9.67	0.47	chromatin	common
ACTR6	A0A0R4J009	9.23	0.06	chromatin	common
ACTR8	Q8R2S9	9.52	0.09	chromatin	common
AHCTF1	Q8CJF7	10.43	-0.25	others	common
AKAP8L	Q9R0L7	10.64	0.86	transcription regulation	common
ALYREF	O08583	11.10	-0.15	RNA processing & translation	common
ARID1A	A2BH40	10.32	1.03	chromatin	common
ARID2	E9Q7E2	9.87	0.17	chromatin	common
ARNTL	Q9WTL8	9.79	-1.22	transcription regulation	common
BANF1	O54962	11.19	0.38	chromatin	common
BAP18	Q9DCT6	9.66	0.72	chromatin	common
BRD4	Q9ESU6	10.61	-0.03	chromatin	common
BRD9	A0A0R4J175	9.84	1.08	chromatin	common
CCDC47	Q9D024	10.02	1.18	others	common
CD2BP2	Q9CWK3	9.93	0.40	RNA processing & translation	common
CDC5L	Q6A068	11.07	-0.73	transcription regulation	common
CENPV	Q9CXS4	11.32	-0.94	chromatin	common
CHD4	Q6PDQ2	11.09	0.69	chromatin	common
CHTOP	Q9CY57	10.68	0.85	transcriptional regulation	common
CLOCK	O08785	9.75	-0.82	transcriptional regulation	common
CMAS	Q99KK2	10.92	-0.47	others	common
CPSF4	E0CXT7	9.79	-0.31	RNA processing & translation	common
CREB1	Q01147	10.24	-0.27	transcription regulation	common
CSTF3	Q99LI7	10.68	-0.26	RNA processing & translation	common
DDOST	O54734	10.43	0.77	membrane	common
DDX17	Q501J6	11.53	0.42	RNA processing & translation	common
DDX23	D3Z0M9	11.13	-1.24	RNA processing & translation	common
DDX5	Q61656	12.05	-0.13	RNA processing & translation	common
DDX50	Q99MJ9	10.24	0.43	RNA processing & translation	common
DNAJA1	P63037	10.57	-0.68	others	common
DNAJA2	Q9QYJ0	10.61	0.37	others	common
DNAJB4	Q9D832	9.69	-1.22	others	common
EED	Q921E6	9.46	-0.95	transcription regulation	common
FARSA	Q8C0C7	9.81	0.27	RNA processing & translation	common
GLE1	Q8R322	9.65	0.30	others	common
GLYR1	Q922P9	10.85	1.24	transcriptional regulation	common
GNL3L	Q6PGG6	9.89	0.48	others	common
GTF2H2	Q9JIB4	9.19	-0.19	transcription regulation	common
GTF3C1	Q8K284	9.88	0.04	transcription regulation	common
GTF3C3	Q3TMP1	9.60	0.70	transcription regulation	common
GTF3C4	Q8BMQ2	9.86	-0.16	transcription regulation	common
H1F0	P10922	11.53	-0.49	histones	common

H2AFX	P27661	11.99	1.00	histones	common
H2AFY	Q9QZQ8	11.59	-0.43	histones	common
H3F3C	P02301	12.43	-0.46	histones	common
HDGFL2	Q3UMU9	10.09	-0.83	chromatin	common
HIST1H1B	P43276	11.09	1.32	histones	common
HIST1H1C	P15864	11.88	-0.63	histones	common
HIST2H2B B	Q64525	12.39	-0.77	histones	common
HIST2H3C 2	P84228	10.88	-0.45	histones	common
HIST4H4	P62806	12.32	-0.72	histones	common
HP1BP3	Q3TEA8	11.40	1.35	chromatin	common
HSD17B12	O70503	9.64	0.08	others	common
INTS1	Q6P4S8	10.19	-0.54	transcription regulation	common
INTS10	Q8K2A7	9.73	-0.34	RNA processing & translation	common
INTS14	Q8R3P6	9.98	-0.50	RNA processing & translation	common
INTS3	Q7TPD0	10.24	-0.01	transcription regulation	common
INTS6	Q6PCM2	9.95	-0.67	RNA processing & translation	common
ISY1	Q69ZQ2	10.21	-0.89	RNA processing & translation	common
KHDRBS1	Q60749	11.52	1.12	RNA processing & translation	common
LAS1L	A2BE28	10.00	0.19	RNA processing & translation	common
LMNA	P48678	12.44	-0.76	membrane associated	common
LMNB1	P14733	12.26	0.99	membrane associated	common
LMNB2	P21619	12.20	1.01	membrane associated	common
LRRC59	Q922Q8	10.50	1.02	others	common
MBNL2	Q8C181-4	10.17	0.47	RNA processing & translation	common
MCM3AP	Q9WUU9	9.95	-0.14	others	common
MED1	Q925J9	9.84	-0.11	transcription regulation	common
MED10	Q9CXU0	9.37	0.49	transcription regulation	common
MED14	A2ABV5	9.76	0.38	transcription regulation	common
MED17	Q8VCD5	9.78	-0.30	transcription regulation	common
MED24	Q99K74	9.71	0.08	transcription regulation	common
MED8	Q9D7W5	9.59	-0.09	transcription regulation	common
MOGS	Q80UM7	9.73	0.33	membrane associated	common
MYL12B	Q3THE2	9.70	0.03	others	common
MYL6	Q60605	10.24	0.45	others	common
NCOA5	Q91W39	11.01	0.96	transcription regulation	common
NONO	Q99K48	12.09	0.13	transcription regulation	common
NOSIP	Q9D6T0	10.22	-0.92	others	common
NR3C1	P06537	9.62	-0.74	transcriptional regulation	common
NRF1	Q3UXF4	9.81	-0.34	transcriptional regulation	common
NSUN2	Q1HFZ0	9.83	-0.21	RNA processing & translation	common
NUP107	Q8BH74	10.71	0.29	membrane associated	common
NUP133	Q8R0G9	10.99	0.28	membrane associated	common
NUP153	E9Q3G8	10.59	-0.38	membrane associated	common
NUP155	Q99P88	10.94	0.05	membrane associated	common
NUP160	Q9Z0W3	10.76	0.20	membrane associated	common
NUP205	B9EJ54	10.86	0.31	membrane associated	common

NUP210	Q9QY81	11.10	-0.26	membrane associated	common
NUP214	Q80U93	10.70	0.24	membrane associated	common
NUP35	Q8R4R6	10.72	0.05	membrane associated	common
NUP37	Q9CWU9	10.63	0.23	membrane associated	common
NUP43	P59235	10.50	0.22	membrane associated	common
NUP54	Q8BTS4	10.94	0.63	membrane associated	common
NUP58	Q8R332	10.35	0.14	membrane associated	common
NUP62	Q63850	10.80	0.46	membrane associated	common
NUP85	Q8R480	10.46	0.17	membrane associated	common
NUP88	Q8CEC0	10.55	-0.05	membrane associated	common
NUP93	Q8BJ71	11.11	0.30	membrane associated	common
NUP98	Q6PFD9	11.15	0.33	membrane associated	common
NXF1	Q99JX7	10.78	-0.71	others	common
PBRM1	Q8BSQ9	10.53	-0.01	chromatin	common
PDS5A	Q6A026	10.26	-0.80	chromatin	common
PDS5B	Q4VA53	10.84	-0.22	chromatin	common
PELP1	Q9DBD5	9.99	0.62	transcription regulation	common
PHF3	B2RQG2	9.83	-0.24	transcription regulation	common
PPIL2	Q9D787	10.12	-0.92	others	common
PRPF3	Q922U1	10.68	-0.65	RNA processing & translation	common
PRPF6	Q91YR7	10.98	-1.25	RNA processing & translation	common
PRPF8	Q99PV0	11.77	-1.18	RNA processing & translation	common
RAD21	Q61550	10.71	-0.67	chromatin	common
RAE1	Q8C570	10.79	0.31	RNA processing & translation	common
RANBP2	Q9ERU9	11.38	0.18	others	common
RBM15	Q0VBL3	10.59	-0.76	RNA processing & translation	common
RBM39	Q8VH51-2	11.16	-0.69	RNA processing & translation	common
RBMXL1	Q91VM5	11.79	0.67	RNA processing & translation	common
RNPC3	Q3UZ01	9.61	-0.19	RNA processing & translation	common
RPL10A	A0A3B2WBL1	10.60	0.61	RNA processing & translation	common
RPL11	Q9CXW4	10.87	0.54	RNA processing & translation	common
RPL12	P35979	10.65	0.17	RNA processing & translation	common
RPL21	O09167	10.74	1.20	RNA processing & translation	common
RPL22	P67984	10.28	0.68	RNA processing & translation	common
RPL23	P62830	10.97	0.56	RNA processing & translation	common
RPL23A	P62751	10.96	0.84	RNA processing & translation	common
RPL27	P61358	10.82	0.77	RNA processing & translation	common
RPL30	P62889	10.51	0.53	RNA processing & translation	common
RPL31	P62900	10.84	0.80	RNA processing & translation	common
RPL35A	O55142	10.65	1.15	RNA processing & translation	common
RPL5	P47962	11.06	0.78	RNA processing & translation	common
RPL9	P51410	10.70	0.60	RNA processing & translation	common
RPN1	Q91YQ5	10.65	1.03	others	common
RPN2	Q9DBG6	10.40	0.80	others	common
RPS11	P62281	10.87	0.80	RNA processing & translation	common
RPS13	P62301	10.63	1.06	RNA processing & translation	common

RPS14	P62264	10.64	0.68	RNA processing & translation	common
RPS15	P62843	10.61	0.75	RNA processing & translation	common
RPS15A	P62245	10.49	0.53	RNA processing & translation	common
RPS16	P14131	10.83	1.25	RNA processing & translation	common
RPS19	Q9CZX8	10.89	1.07	RNA processing & translation	common
RPS2	P25444	10.95	0.99	RNA processing & translation	common
RPS20	P60867	10.59	1.21	RNA processing & translation	common
RPS23	P62267	10.54	0.97	RNA processing & translation	common
RPS25	P62852	10.75	1.31	RNA processing & translation	common
RPS27A	P62983	11.71	-0.39	RNA processing & translation	common
RPS3	P62908	11.11	0.72	RNA processing & translation	common
RPS3A	P97351	11.14	0.69	RNA processing & translation	common
RPS5	Q91V55	10.48	0.36	RNA processing & translation	common
RPS6	P62754	10.71	1.15	RNA processing & translation	common
RPS7	P62082	11.02	0.66	RNA processing & translation	common
RTRAF	Q9CQE8	10.38	1.10	transcription regulation	common
RUVBL1	P60122	11.04	0.03	transcription regulation	common
RUVBL2	Q9WTM5	11.12	0.42	transcription regulation	common
SAFB2	Q80YR5	10.63	-0.84	transcription regulation	common
SART1	Q9Z315	10.87	-0.62	RNA processing & translation	common
SEH1L	Q8R2U0	10.52	0.33	membrane associated	common
SENP3	Q9EP97	9.72	0.71	others	common
SF3B5	Q923D4	10.69	-0.84	RNA processing & translation	common
SFPQ	Q8VIJ6	12.22	0.22	RNA processing & translation	common
SIN3A	Q60520	10.32	0.85	transcription regulation	common
SLTM	Q8CH25-2	10.79	0.80	transcription regulation	common
SMARCA2	Q6DIC0	10.32	0.91	chromatin	common
SMARCA4	Q3TKT4	10.57	0.88	chromatin	common
SMARCA5	Q91ZW3	10.86	-0.18	chromatin	common
SMARCB1	Q9Z0H3	10.22	0.36	chromatin	common
SMARCC1	P97496	9.86	0.31	chromatin	common
SMARCC2	Q3UID0	11.22	0.90	chromatin	common
SMARCE1	O54941	10.69	0.80	chromatin	common
SMC1A	Q9CU62	11.37	-0.88	chromatin	common
SMC3	Q9CW03	11.36	-0.92	chromatin	common
SMCHD1	Q6P5D8	10.60	1.21	chromatin	common
SMPD4	Q6ZPR5	9.99	0.39	membrane associated	common
SNRNP70	Q62376	11.66	-0.99	RNA processing & translation	common
SNRPB	P27048	11.47	-0.77	RNA processing & translation	common
SNRPB2	Q9CQI7	10.83	-0.86	RNA processing & translation	common
SNRPD1	P62315	11.13	0.06	RNA processing & translation	common
SNRPD2	P62317	11.55	-0.62	RNA processing & translation	common
SNRPD3	P62320	11.14	-0.74	RNA processing & translation	common
SNRPF	P62307	10.93	-0.61	RNA processing & translation	common
SNRPG	P62309	11.49	-0.90	RNA processing & translation	common
SNU13	Q9D0T1	11.05	-1.04	RNA processing & translation	common

SNW1	A0A0B4J1E2	10.80	-0.82	transcription regulation	common
SPIN1	Q61142	9.97	0.49	chromatin	common
SSB	P32067	11.44	0.20	other	common
SSR1	A0A286YCT4	10.10	0.46	membrane associated	common
SSRP1	Q08943	10.90	-0.26	chromatin	common
STAG1	Q9D3E6	10.12	-0.72	chromatin	common
STAG2	O35638	10.56	-0.84	chromatin	common
SUB1	P11031	10.41	0.64	transcription regulation	common
SUPT16	G3X956	10.87	-0.23	chromatin	common
TAF1	Q80UV9	9.53	-0.46	transcription regulation	common
TAF6	Q62311	9.76	-0.81	transcription regulation	common
TAF9	Q8VI33	9.75	-0.19	transcription regulation	common
TCERG1	Q8CGF7	11.02	-0.60	transcription regulation	common
TECR	Q9CY27	10.26	0.43	others	common
TERF2	O35144	9.70	1.30	others	common
THOC1	Q8R3N6	10.63	-0.82	RNA processing & translation	common
THOC2	B1AZI6	10.58	-0.90	RNA processing & translation	common
THOC5	A0A0R4J0J6	10.39	-0.68	RNA processing & translation	common
THOC6	Q5U4D9	10.45	-0.58	RNA processing & translation	common
THOC7	Q7TMY4	10.67	-0.38	RNA processing & translation	common
TMPO	Q61033	10.77	0.82	membrane associated	common
TOP1	Q04750	11.05	-0.87	chromatin	common
TOP2B	Q64511	11.15	-0.27	chromatin	common
TRIP12	G5E870	9.98	0.09	others	common
TRRAP	A0A1D5RLL4	10.48	0.35	chromatin	common
UBP1	Q811S7	9.70	0.30	transcription regulation	common
USP39	Q3TIX9	10.66	-0.99	RNA processing & translation	common
WDR18	Q4VBE8	10.38	0.35	others	common
WDR5	P61965	10.33	0.26	chromatin	common
WRNIP1	Q91XU0	10.62	-0.32	others	common
YEATS4	Q9CR11	9.92	0.21	chromatin	common
YTHDC1	E9Q5K9	10.42	0.12	RNA processing & translation	common
ZFP638	A0A0N4SV80	10.76	1.00	transcription regulation	common
ZMYND8	A2A483	10.36	0.23	chromatin	common
ADAR	Q99MU3	10.31	3.82	RNA processing & translation	brain
ADARB1	Q91ZS8-2	10.19	1.76	RNA processing & translation	brain
ADNP	Q9Z103	10.40	1.37	transcription regulation	brain
ATRX	Q61687	10.73	2.10	chromatin	brain
CDK2AP1	O35207	9.84	1.94	others	brain
CHD5	E9PYL1	10.22	3.46	chromatin	brain
ERGIC1	Q9DC16	9.44	2.43	others	brain
FYTTD1	Q91Z49	10.19	1.58	others	brain
GATAD2B	Q8VHR5	10.27	2.27	transcription regulation	brain
GTF2I	Q9ESZ8	10.37	1.67	transcription regulation	brain
HDGFL3	Q9JMG7	10.64	3.46	others	brain
KHDRBS3	Q9R226	11.08	2.15	RNA processing & translation	brain

MECP2	Q9Z2D6-2	11.36	2.97	chromatin	brain
MLF2	Q99KX1	10.41	1.57	transcriptional regulation	brain
MTDH	Q80WJ7	9.93	2.77	transcriptional regulation	brain
NACC1	Q7TSZ8	10.04	1.57	transcriptional regulation	brain
NOMO1	Q6GQT9	10.34	2.13	membrane associated	brain
PSIP1	Q99JF8	11.26	2.77	transcription regulation	brain
PSPC1	Q8R326	11.56	3.22	transcription regulation	brain
RNF14	Q9JI90	10.04	2.05	others	brain
RPL10	Q6ZWV3	10.87	1.61	RNA processing & translation	brain
RPL13	P47963	10.81	1.63	transcription regulation	brain
RPS4X	P62702	11.07	1.42	RNA processing & translation	brain
SERBP1	Q9CY58-2	10.47	1.37	RNA processing & translation	brain
SUGP2	Q8CH09	10.95	4.79	RNA processing & translation	brain
SUN1	Q9D666-3	10.18	2.81	membrane associated	brain
VAPA	Q9WV55	10.34	3.11	membrane associated	brain
ZMYM3	Q9JLM4	9.35	2.03	transcription regulation	brain
ARGLU1	Q3UL36	11.01	-1.57	others	liver
CACTIN	Q9CS00	9.77	-1.83	others	liver
DDX21	Q9JIK5	10.86	-3.08	transcription regulation	liver
DEK	Q7TNV0	11.05	-2.12	chromatin	liver
DKC1	Q9ESX5	10.57	-2.33	RNA processing & translation	liver
H2AFZ	P43274	11.49	-1.38	histones	liver
HIST1H1E	P0C0S6	11.12	-1.64	histones	liver
IK	Q9Z1M8	10.86	-1.35	RNA processing & translation	liver
LENG8	D3YWS8	9.27	-2.15	others	liver
LIG3	P97386	9.98	-1.49	others	liver
MYBBP1A	Q7TPV4	11.16	-1.83	transcription regulation	liver
MYH9	Q8VDD5	10.18	-1.75	others	liver
NFIX	E9PUH7	10.48	-1.37	transcription regulation	liver
NMNAT1	Q9EPA7	10.00	-2.20	others	liver
NOP56	Q9D6Z1	10.90	-1.79	RNA processing & translation	liver
NOP58	Q6DFW4	10.80	-1.74	RNA processing & translation	liver
PCMTD2	B0R0C7	10.26	-2.45	others	liver
PNKP	G5E8N7	10.15	-1.85	others	liver
PUM3	Q8BKS9	10.25	-1.87	others	liver
RCC1	Q8VE37	10.59	-1.32	chromatin	liver
RRP12	Q6P5B0	10.02	-1.34	RNA processing & translation	liver
RSL1D1	Q8BVY0	10.40	-1.62	RNA processing & translation	liver
SMARCD2	Q99JR8	10.01	-2.24	chromatin	liver
SNRPA1	P57784	11.14	-1.38	RNA processing & translation	liver
SON	H9KV00	11.47	-1.36	RNA processing & translation	liver
TFIP11	Q9ERA6	10.60	-1.32	RNA processing & translation	liver
TOR1AIP1	Q921T2	10.52	-2.19	membrane associated	liver
ZNF281	Q99LI5	9.95	-1.30	transcription regulation	liver

Filtered protein list obtained from the MaxQuant search against the UniProt database for *Mus musculus*. BC: brain PCH; LC: liver PCH. Protein function and enrichment category according to the classification in Figure S3.
